# Synchronous Differentiated Thyroid Carcinoma in Non-Invasive Follicular Thyroid Neoplasm with Papillary-like Nuclear Features (NIFTP): Prevalence, Pathological Profile, and Limits of Pre-Operative Risk Prediction in a Multicentre Cohort

**DOI:** 10.3390/cancers18213505

**Published:** 2026-10-30

**Authors:** Chiara Dobrinja, Manuela Mastronardi, Stella Bernardi, Elena Bonati, Marco Bononi, Pietro Giorgio Calò, Gian Luigi Canu, Federico Cappellacci, Maria Grazia Chiofalo, Loredana De Pasquale, Carmela De Crea, Marco Stefano Demarchi, Paolo Del Rio, Giacomo Di Filippo, Giovanni Docimo, Daniele Gasparini, Rita Gervasi, Giuseppa Graceffa, Angela Gurrado, Maurizio Iacobone, Eduardo Mercelo Iervolino, Nadia Innaro, Rita Laforgia, Giovanni Lazzari, Petra Makovac, Gabriele Materazzi, Fabio Medas, Eleonora Morelli, Barbara Mullineris, Piermarco Papini, Francesco Pennestrì, Angela Pezzolla, Marco Raffaelli, Esther Diana Rossi, Leonardo Rossi, Gregorio Scerrino, Luca Sessa, Mario Testini, Emanuela Traini, Nicolò de Manzini, Dario Gregori

**Affiliations:** 1 UCO Clinica Chirurgica, Department of Medicine, Surgery and Health Sciences, University of Trieste, Azienda Sanitaria Universitaria Giuliano Isontina (ASUGI), Cattinara Teaching Hospital, 34149 Trieste, Italy; manuela.mastronardi@units.it (M.M.); ndemanzini@units.it (N.d.M.); 2 SS Endocrinologia, Department of Medicine, Surgery and Health Sciences, University of Trieste, Azienda Sanitaria Universitaria Giuliano Isontina (ASUGI), Cattinara Teaching Hospital, 34149 Trieste, Italy; stella.bernardi@units.it; 3 Unit of General Surgery, Department of Medicine and Surgery, University Hospital of Parma, 43126 Parma, Italy; elena.bonati@unipr.it (E.B.); paolo.delrio@unipr.it (P.D.R.); 4 DAI Chirurgia Generale, Chirurgia Plastica e Ortopedia, Policlinico Umberto I, Sapienza University of Rome, 00161 Rome, Italy; marco.bononi@uniroma.it; 5 Department of Surgical Sciences, University of Cagliari, Monserrato, 09042 Cagliari, Italy; pgcalo@unica.it (P.G.C.); gianl.canu@unic.it (G.L.C.); f.cappellacci@aoucagliari.it (F.C.); fabiomedas@unica.it (F.M.); 6 SC Chirurgia Oncologica della Tiroide, Istituto Nazionale Tumori, IRCCS Fondazione G. Pascale, 80131 Naples, Italy; m.chiofalo@istitutotumori.na.it; 7 Thyroid Unit, ASST Santi Paolo e Carlo, San Paolo University Hospital, 20142 Milan, Italy; loredana.depasquale@asst-santipaolocarlo.it; 8 Ospedale Isola Tiberina, Gemelli Isola, Centro di Ricerca in Chirurgia delle Ghiandole Endocrine e dell’Obesità, Università Cattolica del Sacro Cuore, 00186 Rome, Italy; carmela.decrea@unicatt.it; 9 Department of Thoracic and Endocrine Surgery, Faculty of Medicine, University Hospitals of Geneva, Rue Gabrielle-Perret-Gentil 4, 1205 Geneva, Switzerland; marcostefano.demarchi@hug.ch; 10 Endocrine Surgery Unit, Department of Surgery and Oncology, Verona University Hospital, P.le L.A. Scuro 10, 37134 Verona, Italy; giacomo.difilippo@aovr.veneto.it (G.D.F.); giovanni.lazzari@aovr.veneto.it (G.L.); eleonore.morelli@aovr.veneto.it (E.M.); 11 Unità Operativa Semplice Dipartimentale di Chirurgia Tiroidea, Azienda Ospedaliera Universitaria “Luigi Vanvitelli”, 80138 Naples, Italy; giovanni.docimo@unicampania.it (G.D.); eduardomarcelo.iervolino@policliniconapoli.it (E.M.I.); 12 Unit of Biostatistics, Epidemiology and Public Health, University of Padova, 35131 Padova, Italy; daniele.gasparini@ubep.unipd.it (D.G.); dario.gregori@unipd.it (D.G.); 13 UOC Endocrinochirurgia, AOU Dulbecco, 88100 Catanzaro, Italy; ritage@email.it (R.G.); nadia.innaro@aourenatodulbecco.it (N.I.); 14 Dipartimento MEPRE-J, Chirurgia Oncologica, Policlinico P. Giaccone, Università degli Studi di Palermo, 90127 Palermo, Italy; giuseppa.graceffa@unipa.it; 15 UOC Chirurgia Generale Universitaria “V. Bonomo”, Dipartimento di Medicina di Precisione e Rigenerativa e Area Jonica (DiMePRe-J), Università degli Studi di Bari, 70124 Bari, Italy; angela.gurrado@uniba.it (A.G.); mario.testini@uniba.it (M.T.); 16 Endocrine Surgery Unit, Department of Surgery, Oncology and Gastroenterology, University of Padova, 35128 Padova, Italy; maurizio.iacobone@unipd.it; 17 Laparoscopic and Emergency General Surgery Unit, Section of Surgery, Department of Precision and Regenerative Medicine and Jonic Area (DiMePRe-J), Hospital University of Bari, 70124 Bari, Italy; ritalaforgia@hotmail.it (R.L.); angela.pezzolla@uniba.it (A.P.); 18 Servizio di Chirurgia, Ospedale Regione di Mendrisio—OBV, EOC, Via Alfonso Turconi 23, 6850 Mendrisio, Switzerland; petra.makovac@eoc.ch; 19 Department of Surgical, Medical and Molecular Pathology and Critical Area, University of Pisa, 56126 Pisa, Italy; gabriele.materazzi@unipi.it (G.M.); piermarcopapini@gmail.com (P.P.); leonardo.rossi1@unipi.it (L.R.); 20 Department of General, Emergency and New Technologies, University Hospital of Modena, Baggiovara Civil Hospital, 41126 Modena, Italy; mullineris.barbara@aou.mo.it; 21 UOC Chirurgia Endocrina e Metabolica, Centro Dipartimentale di Chirurgia Endocrina e dell’Obesità, Fondazione Policlinico Universitario Agostino Gemelli IRCCS, 00168 Rome, Italy; francesco.pennestri@policlinicogemelli.it (F.P.); marco.raffaelli@policlinicogemelli.it (M.R.); esther.rossi@policlinicogemelli.it (E.D.R.); 22 Unit of Endocrine Surgery, Department of Surgical Oncological and Oral Sciences, Policlinico “P. Giaccone”, University of Palermo, Via Liborio Giuffré 5, 90127 Palermo, Italy; gregorio.scerrino@tiscali.it; 23 Division of Endocrine Surgery, Saint Camillus International University of Health Sciences (UniCamillus), 00131 Rome, Italy; luca.sessa@unicamillus.it; 24 UOC Chirurgia Endocrina, Ospedale San Carlo di Nancy, 00165 Rome, Italy; etraini@gvmnet.it; 25 BIOSTAT-X, Institute of Pediatric Research (IRP) “Città della Speranza”, 35127 Padova, Italy

**Keywords:** NIFTP, thyroid neoplasms, papillary microcarcinoma, synchronous DTC, clinical prediction model, internal–external validation, machine learning, thyroid surgery

## Abstract

**Background/Objectives:** Non-invasive follicular thyroid neoplasm with papillary-like nuclear features (NIFTP) was reclassified as a non-malignant entity in 2016 to reduce overtreatment. A proportion of NIFTP-containing glands nonetheless harbour a synchronous differentiated thyroid carcinoma (DTC). We quantified its prevalence and pathological profile and assessed whether routinely collected pre-operative variables identify affected patients. **Methods:** A multicentre retrospective cohort of 1185 adults with NIFTP confirmed at second histological review, operated on between 2017 and 2023 in 22 centres. The outcome was a synchronous DTC in the surgical specimen. A weighted random forest and an elastic-net logistic regression based on 13 pre-operative predictors were evaluated with 20-times repeated five-fold cross-validation, with all data-driven steps nested within training folds, and with leave-one-centre-out and temporal validation. **Results:** A synchronous DTC was present in 61 patients (5.1%; 95% CI 4.0–6.6; 6.2% after review of the stored reports), with marked between-centre variation (none recorded in eight centres). Fifty-eight carcinomas (95%) were pT1a; none had gross extra-thyroidal extension or nodal metastasis (nodes examined in 27 patients), and none met the 2025 American Thyroid Association criteria mandating a more extensive operation. No pre-operative variable differed between groups after multiplicity adjustment. The cross-validated AUROC was 0.56 (95% CI 0.47–0.65) for the random forest and 0.58 (0.48–0.67) for the elastic net; the within-centre concordance was 0.48 and the temporal-validation AUROC 0.39, and decision-curve analysis showed no net benefit over default strategies. **Conclusions:** About one in twenty NIFTP-containing specimens harbours a synchronous DTC, usually a low-risk papillary microcarcinoma. Routinely available pre-operative variables do not identify these patients; management should be guided by the pathology of the carcinoma itself.

## Simple Summary

NIFTP is a thyroid lesion that was reclassified about ten years ago as non-cancerous, so that patients would not be treated as if they had cancer. In some patients, however, a true thyroid cancer is found elsewhere in the same gland after operation. In this study of 1185 patients treated in 22 surgical centres, about one in twenty patients with NIFTP also had a thyroid cancer, usually a papillary microcarcinoma of 1 cm or less confined to the gland; on the available pathology, none of the cancers recorded by the centres had features that would require a more extensive operation according to current guidelines. We also asked whether information available before surgery—such as ultrasound, cytology, blood tests, and body weight—could identify these patients in advance. When tested rigorously, including across centres, it could not. Therefore, decisions concerning further surgery should rest on the pathological features of the carcinoma found, not on a pre-operative risk estimate.

## 1. Introduction

Thyroid cancer is the most common endocrine malignancy worldwide, and its incidence has increased substantially over the past decades, in large part owing to the heightened detection of small, indolent tumours [1]. Against this background, in 2016 a landmark multinational study proposed reclassifying the encapsulated, non-invasive follicular variant of papillary thyroid carcinoma (EFVPTC) as a non-malignant entity, introducing the term “non-invasive follicular thyroid neoplasm with papillary-like nuclear features” (NIFTP) [2]. The reclassification was explicitly intended to spare patients unnecessary total thyroidectomy and radioactive-iodine therapy, to reduce the psychological burden of a “carcinoma” label in lesions with an exceedingly favourable prognosis, and to limit overtreatment [2,3,4]. NIFTP has since been formally incorporated into the 2022 World Health Organization (WHO) classification of thyroid lesions, where it is placed among the low-risk follicular-cell-derived lesions [5,6], and conservative management of isolated NIFTP is endorsed by current American Thyroid Association (ATA) recommendations [7,8].

NIFTP is currently defined as a completely encapsulated or clearly demarcated follicular-patterned thyroid neoplasm displaying papillary thyroid carcinoma-like nuclear features in the absence of capsular or vascular invasion [5,9]. Diagnostic confirmation requires exhaustive histological examination of the entire tumour capsule; because invasion may be focal, incomplete sampling can lead to the misclassification of an invasive encapsulated follicular-variant papillary thyroid carcinoma as NIFTP [10,11]. Interobserver variability in the assessment of the nuclear score remains non-trivial even among expert thyroid pathologists [12,13], and reported NIFTP frequencies vary widely across geographical regions and over time, reflecting both genuine biological differences and the progressive adoption of stricter criteria: a meta-analysis of 50 studies and more than 100,000 papillary carcinomas found a pooled NIFTP incidence of 6.0%, ranging from 9.6% in Europe and 9.3% in North America to 2.1% in Asia [14,15,16].

Critically, NIFTP remains a post-operative, histological diagnosis. Neither cytology nor ultrasonography reliably separates NIFTP from invasive follicular-patterned carcinoma or from follicular adenoma before surgery [17,18,19,20]. Most NIFTP lesions fall into the indeterminate categories of the Bethesda and TIR systems, and a minority are reported as suspicious or malignant, so that the introduction of NIFTP has measurably altered the apparent risk of malignancy of indeterminate cytology [21,22,23,24]. At the molecular level, NIFTP is characteristically RAS-like, with enrichment for NRAS, HRAS, and KRAS alterations and occasional THADA–IGF2BP3 or PAX8–PPARG fusions, whereas BRAF V600E is generally regarded as an exclusion criterion [25,26,27,28]. Recent work nonetheless suggests that a subset of lesions with a nuclear score of 3, and a proportion of cases positive for BRAF V600E on immunohistochemistry, may represent misclassified invasive tumours rather than true NIFTP, and should prompt molecular work-up [29,30,31].

Despite its reclassification as non-malignant, NIFTP retains clinical significance because a non-negligible proportion of patients have a synchronous differentiated thyroid carcinoma (DTC) elsewhere in the gland [32,33,34]. These carcinomas—most commonly papillary thyroid microcarcinoma—are typically discovered during an intraoperative frozen section of a separate nodule or, more often, only at definitive histology [35,36]. The reported co-occurrence varies substantially across series, and its determinants are poorly understood [37,38]. Rare reports of nodal metastasis in cases labelled NIFTP—almost invariably in patients with a synchronous DTC—have further fuelled the debate on how much diagnostic and surgical caution is warranted [39,40]. The presence of a synchronous DTC alongside NIFTP raises practical questions regarding the extent of thyroidectomy, the need for completion surgery, radioactive-iodine administration, and long-term follow-up, because management should be dictated by the biology and stage of the synchronous DTC rather than by the NIFTP itself [9,41]. Whether pre-operative information can anticipate a synchronous DTC is, therefore, a recurrent question, but two features constrain how it can be asked. First, NIFTP is itself a histological diagnosis, so that any pre-operative estimate is necessarily conditional on a final diagnosis that is unknown when surgery is planned. Second, an anticipated synchronous DTC is clinically consequential only if its size, stage, or histotype would change the extent of surgery under contemporary guidelines, in which lobectomy is acceptable for most low-risk intrathyroidal carcinomas and active surveillance is an option for papillary microcarcinoma [8,42,43,44].

Traditional logistic regression remains the workhorse for clinical risk prediction; machine-learning algorithms may offer advantages in high-dimensional or non-linear settings and have been applied to the pre-operative characterisation of thyroid nodules [45,46,47]. Random forests combine bootstrap aggregation with random feature selection at each split, accommodating missing data and complex interactions [45,48]. For imbalanced classification problems—where the event of interest is rare—extensions such as weighted random forests and random-forest quantile classifiers have been proposed [49,50], and evaluation at the default 0.50 probability threshold typically yields near-perfect specificity at the cost of negligible sensitivity, so that thresholds are often chosen by maximising a balanced criterion such as the Youden index [51,52]. Corrections for class imbalance, however, distort predicted probabilities [53], and the apparent performance of flexible algorithms is particularly vulnerable to optimism when events are few and data are clustered by centre. Nesting every data-driven step within the validation procedure and assessing performance across centres are therefore required before any claim of clinical usefulness [54,55,56].

The primary objective of this study was to quantify the prevalence and the pathological profile of synchronous DTC in a large multicentre cohort of patients with histologically proven NIFTP, with particular attention to whether the synchronous DTCs would alter surgical management under current guidelines. The secondary objective was to determine whether routinely collected pre-operative clinical, ultrasonographic, cytological, and biochemical variables discriminate, among patients whose nodule proved to be NIFTP, those with a synchronous DTC. To this end, we (i) compared pre-operative features between groups, (ii) developed a weighted random forest and a penalised logistic regression and evaluated both with fully nested repeated cross-validation, (iii) assessed transportability across centres and over time, and (iv) quantified calibration and clinical utility by decision-curve analysis. Reporting follows the TRIPOD+AI, TRIPOD-Cluster, and STROBE recommendations [56,57,58].

## 2. Materials and Methods

### 2.1. Study Design and Population

**Figure 1:**
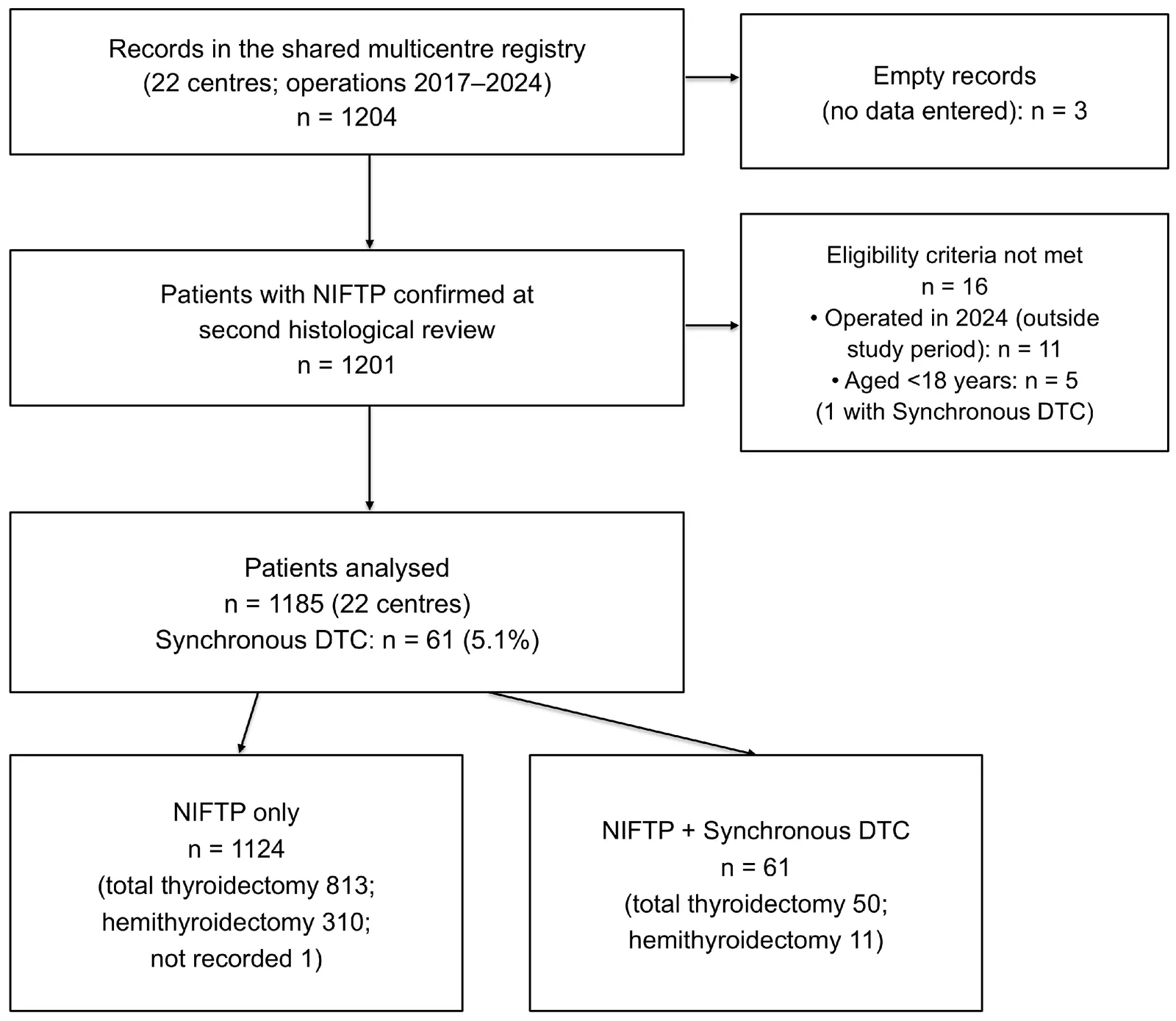
Flow of registry records and patients. DTC, differentiated thyroid carcinoma; NIFTP, non-invasive follicular thyroid neoplasm with papillary-like nuclear features.

We conducted a multicentre retrospective cohort study based on a shared registry to which 22 endocrine-surgery referral centres contributed all consecutive adult patients (aged 18 years or older) with a histologically proven diagnosis of NIFTP who underwent thyroid surgery between 1 January 2017 and 31 December 2023. Data were extracted retrospectively from clinical records by each centre into a common case-report form. The registry did not record the total number of thyroidectomies performed at each centre during the study period, so that a screening denominator is not available. Of 1204 registry records, 3 were empty and 16 did not meet the eligibility criteria (11 operations performed in 2024 and 5 patients younger than 18 years), leaving 1185 patients for analysis (Figure 1). Centre-specific enrolment, period of recruitment, extent of surgery, outcome prevalence, and data completeness are reported in Supplementary Table S4, where centres are identified by code.

Each patient contributed a single surgical specimen, that of the index operation (hemithyroidectomy or total thyroidectomy); specimens from completion surgery were not counted as separate observations, and all proportions in this report therefore refer equally to patients and to index specimens. The primary outcome was the presence of a synchronous DTC in the index specimen, recorded as a binary variable separately from the NIFTP lesion. Because a carcinoma can be found only in the tissue that is removed, ascertainment is complete after total thyroidectomy and limited to the resected lobe after hemithyroidectomy; this was addressed in a sensitivity analysis. Because the outcome was coded by the centres, we also reviewed the stored reports of the NIFTP-only patients for a separately described carcinoma. This was possible only for the reports that explicitly named NIFTP, since the others described the index lesion in the terminology of the original diagnosis; carcinomas identified in this way were counted as synchronous DTCs in a sensitivity analysis (adjudicated outcome) and in the strict-histology subset, whereas the primary analysis retained the coding of the centres. The original protocol foresaw a minimum post-operative follow-up of 24 months; follow-up information was, however, recorded for only 208 patients. Since the outcome is determined on the surgical specimen, follow-up was not used as an eligibility criterion and no follow-up outcome is reported.

### 2.2. Histopathological Assessment

All cases underwent secondary review by an additional pathologist at each participating centre, blinded to the original report, who retrospectively applied the histological criteria endorsed by the 2022 WHO classification and reiterated in the 2025 ATA guidelines [5,8]: (A) encapsulation or clear demarcation; (B) follicular growth pattern, including <1% or absence of papillae, absence of psammoma bodies and <30% solid, trabecular or insular growth; (C) nuclear features of papillary thyroid carcinoma; (D) no invasive characteristics (no capsular and no vascular invasion); (E) no tumour necrosis; and (F) no high mitotic activity, defined as <3 mitoses per 10 high-power fields. To be eligible, the lesion had to be sampled in its entirety, since focal invasion may otherwise be missed [10,12]. A second review was performed at the participating centres and not by a single central panel. For 491 patients (41%), the free-text histological report stored in the registry did not mention NIFTP and used the terminology of the original report (most often follicular-variant papillary carcinoma, or a TNM category only); for these patients, the NIFTP classification rests on the second-review diagnosis coded in the registry, which could not be re-verified against the slides. A sensitivity analysis was therefore restricted to a strict-histology subset of patients whose stored report explicitly named NIFTP, whose coded and free-text histological fields reported no feature incompatible with NIFTP for the index lesion, and who had no BRAF V600E mutation on pre-operative cytology (*n* = 680; definition in Supplementary Section S1.10).

Features of the NIFTP lesion and of the synchronous DTC were recorded in separate fields: for the carcinoma, histotype and subtype, maximum diameter of the largest focus, multifocality, and stage. Microscopic features (capsular invasion, angio-invasion, lymphatic invasion, tumour necrosis, and extra-thyroidal extension) were recorded at the specimen level. In specimens coded as NIFTP-only, these features refer only to the index lesion, unless a separate carcinoma was present but not coded; in specimens with a synchronous DTC, they were attributed to the carcinoma after case-level review of the registry fields and of the stored report (Supplementary Table S7). Synchronous DTCs were staged according to the eighth edition of the AJCC/TNM system on the basis of the largest focus, with minimal extra-thyroidal extension not being T-defining [59]; where the stage recorded by the centre was inconsistent with the recorded diameter, the size-derived category was used and the discordance is reported. Nodal status was classified as pN0 when at least one lymph node had been examined and found free of metastasis, and as pNX otherwise. A synchronous DTC was considered to mandate a more extensive operation if it was larger than 4 cm or showed gross extra-thyroidal extension or nodal or distant metastasis [8], and to be of potential management relevance if it was larger than 1 cm or showed minimal extra-thyroidal extension, vascular invasion, or an aggressive subtype such as tall-cell [7,8].

### 2.3. Pre-Operative Variables

Ultrasonographic risk was categorised via the European Thyroid Imaging Reporting and Data System (EU-TIRADS) [60,61], and cytology was classified according to the Italian consensus (TIR) categories, with correspondence to the Bethesda system [24,62]. Fourteen pre-operative variables were selected a priori as candidate predictors on the basis of clinical relevance and prior literature: sex, age, pre-operative clinical diagnosis, EU-TIRADS category, cytology category, echogenicity, vascularisation pattern, sonographic margins, nodule diameter on ultrasound (mm), body-mass index (BMI), family history of thyroid disease, serum calcitonin, anti-thyroglobulin antibodies (Ab-Tgs), and anti-thyroid-peroxidase antibodies (Ab-TPOs). Their definitions, allowable values, coding, reference categories, and harmonisation are reported in Supplementary Table S5. In brief, the pre-operative clinical diagnosis was the indication for surgery recorded by the treating team (goitre or multinodular goitre, indeterminate nodule, benign nodule, or nodule suspicious for malignancy on clinical, ultrasonographic, or cytological grounds); EU-TIRADS (categories 4A and 4B, used by one centre, harmonised to 4) and cytology were treated as ordinal; echogenicity (hypoechoic versus other), vascularisation (central and peripheral versus peripheral or absent) and margins (irregular versus regular) were dichotomised, the case-report-form code ‘not determined’ being treated as missing; and Ab-Tgs, Ab-TPOs and calcitonin were recorded by the centres as positive (elevated) or negative according to local laboratory reference ranges, titres not being available. Calcitonin was elevated in only 4 of 395 patients with a measurement, none of whom had a synchronous DTC, and was therefore excluded from modelling as a near-constant variable, so that 13 predictors (16 parameters) were modelled.

Ultrasound, cytology, and molecular results refer to the dominant nodule that prompted surgery, as recorded in the registry; the registry did not link them explicitly to the lesion eventually diagnosed as NIFTP or carcinoma, and their correspondence was examined indirectly by comparing pre-operative and pathological diameters. Post-operative variables (capsular or angio-invasion, multifocality, pathological diameter, extra-thyroidal extension) and intraoperative decisions (extent of resection, lymph-node dissection) were excluded from the models to prevent look-ahead bias. The intended time of prediction is the completion of the pre-operative work-up. Because NIFTP is diagnosed only after surgery, the analysis addresses whether, among patients whose operated nodule proves to be NIFTP, the pre-operative information discriminates those with a synchronous DTC; the population to which such a model could be applied prospectively is not identifiable before surgery, a point taken up in the Discussion.

### 2.4. Statistical Analysis

Categorical variables are summarised as counts (percentages) of patients with available data and were compared with Fisher’s exact test; continuous variables are summarised as the median (first quartile, third quartile) and were compared using the Wilcoxon rank-sum test. *p*-values for the 16 comparisons in Table 1 were adjusted via the Benjamini–Yekutieli procedure, which controls the false discovery rate under arbitrary dependence among tests; adjusted values are reported as q-values [63]. Prevalence is reported with Wilson 95% confidence intervals (CIs). Heterogeneity across centres was described with a random-intercept logistic model, and temporal trends in prevalence and in the extent of surgery with random-intercept logistic models, including year of surgery.

*Sample size and model complexity.* The study analysed all eligible registry patients. Following Riley et al. [64,65], a model with 10 parameters, an outcome prevalence of 5.1%, and an anticipated C-statistic of 0.70 (Cox–Snell R^2^ 0.026) would require at least 3412 patients (176 events) to target a global shrinkage factor of 0.9 or more. With 1185 patients and 61 events, the expected shrinkage for the 16 candidate parameters was 0.66, and only about two to six parameters (1.9–5.7, depending on the anticipated C-statistic) could be supported (Supplementary Section S1.7). Model development was therefore regarded as exploratory, and the emphasis was placed on an internal validation procedure able to reveal optimism [66,67,68].

*Models.* Two algorithms were fitted. The primary model was a weighted random forest (randomForestSRC [69]; AUC-based splitting with 10 random split points; 1000 trees per imputed dataset; class weights inversely proportional to the outcome frequencies in the training data) [45,49,50]; the number of candidate variables per split (2, 3, or 5) and the minimum terminal-node size (3, 10, or 30) were tuned by maximising the out-of-bag AUROC within the training data. The benchmark was a penalised logistic regression with elastic-net regularisation (mixing parameter α = 0.5; λ selected by ten-fold cross-validation within the training data with the one-standard-error rule; balanced observation weights) [70,71].

*Missing data.* Missing predictor values were handled by multiple imputation by chained equations (five imputations, ten iterations; predictive mean matching for continuous and ordinal variables, logistic regression for binary and polytomous regression for nominal variables; all other predictors in the imputation model) [72,73]. The imputation model was fitted on the training data only and then used to impute the held-out patients, and the outcome was excluded from it, so that predictions for held-out patients were obtained as they would be for a new patient [74]. Each model was fitted to each imputed training set, and predicted probabilities were averaged across imputations.

*Internal validation.* Performance was estimated with 20-times repeated, outcome-stratified five-fold cross-validation, in which every data-driven step—imputation, hyperparameter tuning, class weighting, selection of λ, and selection of the classification threshold—was repeated within each training partition and the resulting rule was applied once to the corresponding held-out fold [75,76]. The threshold was the one maximising the Youden index on predictions internal to the training partition (out-of-bag predictions for the random forest and inner cross-validated predictions for the elastic net) [51,52]. Within each repeat, out-of-fold predictions for all patients were pooled to compute performance measures; estimates were then combined across the 20 repeats with Rubin’s rules, the total variance being the average within-repeat variance (DeLong for the AUROC, 200 bootstrap replicates otherwise) plus the between-repeat variance multiplied by (1 + 1/20), on the logit scale for proportions and the AUROC, on the log scale for ratios, and on the original scale for the other measures [77,78].

*Performance measures.* Discrimination was assessed with the AUROC and with the area under the precision–recall curve (average precision), whose no-skill reference equals the outcome prevalence [79]; overall accuracy with the Brier score [80]; and calibration with calibration-in-the-large, the calibration slope, and the expected-to-observed (E/O) ratio [81,82]. At the nested threshold we report the full confusion matrix, sensitivity, specificity, positive and negative predictive values, and likelihood ratios. The random forest and the elastic net were compared within each repeat with the paired DeLong test, and the differences were pooled as above [77].

*Decision-curve analysis.* Clinical utility was assessed by decision-curve analysis [83,84,85,86]. A positive classification was defined as a recommendation, made before surgery, of total thyroidectomy rather than lobectomy as the initial operation; the benefit is the avoidance of completion surgery in a patient with a synchronous DTC, and the harm an unnecessary contralateral lobectomy. Net benefit was computed as TP/*n* − FP/*n* × *p*_t_/(1 − *p*_t_), where TP and FP are the numbers of true- and false-positive classifications and *p*_t_ is the threshold probability, and is reported with 95% CIs (pooled as above) for *p*_t_ between 1% and 30%. The 5–20% range corresponds to accepting between 19 and 4 unnecessary contralateral lobectomies to anticipate one synchronous DTC, which we consider a plausible range of clinical preferences given the low risk of most synchronous DTCs and the morbidity of completion surgery.

*Centre-aware and temporal validation.* Transportability was assessed by internal–external cross-validation, in which each centre was held out in turn and the complete pipeline was developed on the remaining 21 centres [55,87]. Pooled leave-one-centre-out performance is reported together with centre-specific AUROCs (centres with at least three patients with and without the outcome) and observed-to-expected ratios, summarised by random-effects meta-analysis (restricted maximum likelihood) on the logit and log scales with 95% prediction intervals [88]. Within-centre discrimination was summarised by the centre-stratified concordance statistic of the cross-validated predictions, with a cluster-bootstrap 95% CI. For temporal validation, the pipeline was developed on patients operated on in 2017–2020 and evaluated on those operated on in 2021–2023.

*Contribution of individual predictors.* The permutation importance native to randomForestSRC permutes a predictor among the out-of-bag cases of each tree without refitting the model or altering the outcome, and is biassed in favour of correlated predictors [89,90]. We therefore first examined the Spearman correlation matrix of the predictors and then quantified each predictor’s contribution in three ways: (i) held-out permutation importance, the decrease in the cross-validated AUROC when the predictor was randomly permuted in the held-out fold while the fitted model and the outcome were left unchanged; (ii) leave-one-covariate-out (LOCO) importance, the decrease in the cross-validated AUROC when the complete pipeline was refitted without the predictor [91]; and (iii) the randomForestSRC permutation importance (Brier-score-based) computed in each of the 100 training forests. Uncertainty is expressed by the distribution across repeats or training partitions. The shape of the association of continuous and ordinal predictors with predicted risk was described by partial-dependence functions computed in each training partition, with 95% bands across the 100 partitions. These analyses describe the fitted models and do not estimate causal or epidemiological associations [92].

*Sensitivity analyses.* The pipeline was repeated (10 repeats each) (i) with the native on-the-fly imputation of randomForestSRC nested within training partitions, (ii) including the outcome in the imputation of the training data, held-out patients still being imputed without it [93], (iii) with missing-indicator coding, which allows missingness itself to act as a predictor, (iv) with a reduced prespecified model restricted to the eight predictors with less than 30% missing values, (v) without class weighting, (vi) in patients who underwent total thyroidectomy, (vii) in the 14 centres with at least one recorded synchronous DTC, (viii) with the adjudicated outcome, and (ix) in the strict-histology subset. The cross-validated predictions of the primary model were also evaluated against the restricted outcome of a synchronous DTC of potential management relevance. Finally, the analysis reported in the original submission was reproduced and its design elements were modified one at a time (Supplementary Section S3). All analyses were performed in R version 4.3.3 (R Foundation for Statistical Computing, Vienna, Austria) [94] with randomForestSRC 3.4.5, glmnet 4.1-8, mice 3.16.0, pROC 1.18.5, metafor 4.4-0, lme4 1.1-35.2, and pmsampsize 1.1.3; the complete pipeline is described in Supplementary Section S1.

## 3. Results

### 3.1. Cohort and Prevalence of Synchronous DTC

Of 1204 registry records, 1185 patients from 22 centres met the eligibility criteria (Figure 1); centre size ranged from 1 to 289 patients (Supplementary Table S4). A synchronous DTC was identified in 61 patients (5.1%; 95% CI 4.0–6.6); the remaining 1124 had NIFTP without a synchronous DTC. Recorded prevalence differed markedly across centres (*p* < 0.001). No synchronous DTC was recorded in 8 centres contributing 591 patients (50%), including the two largest (289 and 261 patients), whereas in the 14 centres with at least one recorded case, the prevalence was 10.3% (61/594), ranging from 1.1% to 31.6% among centres with at least 15 patients. In a random-intercept model, the between-centre standard deviation on the logit scale was 1.79 (intraclass correlation 0.49); the median centre prevalence was 5.4% (95% CI 2.2–12.7) and the 95% prediction interval for the prevalence in a new centre was 0.2–65%. Prevalence did not change over the study period (odds ratio per year 0.97, 95% CI 0.81–1.17), whereas the proportion of total thyroidectomies decreased (odds ratio per year 0.86, 0.79–0.94). A synchronous DTC was found in 50 of 863 patients (5.8%) after total thyroidectomy and in 11 of 321 (3.4%) after hemithyroidectomy (*p* = 0.11). Review of the 637 stored reports of NIFTP-only patients that named NIFTP identified 13 that described a separate carcinoma not coded as a synchronous DTC (Supplementary Table S7): 12 papillary microcarcinomas of 4 mm or less (reported as ‘papillary microtumour’ in 7), all from one centre that had coded 9 other cases, and one encapsulated angio-invasive follicular carcinoma of 47 mm, treated by total thyroidectomy. With these cases, the prevalence was 6.2% (74 of 1185; 95% CI 5.0–7.8). In the strict-histology subset, 66 of 680 patients (9.7%) had a synchronous DTC after review of the reports (54 as coded by the centres).

The pre-operative characteristics of the cohort are summarised in Table 1. No pre-operative variable differed significantly between groups after adjustment for multiplicity (all *q* ≥ 0.95). BRAF V600E was detected on pre-operative cytology in 33 of 589 tested patients in the NIFTP-only group, 31 of them from a single centre, and in 2 of 27 with a synchronous DTC. Missing data were frequent and strongly centre-dependent (Supplementary Table S6 and Figure S1): the proportion of missing values ranged from 0.1% for the pre-operative diagnosis to 69% for Ab-TPOs, centre membership accounted for much of the variation in missingness (McFadden R^2^ 0.20–0.71), and BMI, family history, and calcitonin were more often recorded in patients with a synchronous DTC, also within centres; antibody results showed the same pattern, less precisely after adjustment for centre. The registry does not record why a value is missing.

### 3.2. Clinical and Pathological Profile

The median age was 52 years in the NIFTP-only group and 55 years in patients with a synchronous DTC; about one in four patients were male in both groups. Total thyroidectomy was the initial procedure in 72% of the NIFTP-only group and in 82% of patients with a synchronous DTC.

Pathological characteristics are reported in Table 2 and, case by case, in Supplementary Table S7. The synchronous DTC was a papillary carcinoma in 57 of 61 cases (93%; classic subtype 6, follicular subtype 3, tall-cell subtype 1, subtype not specified 47), a follicular carcinoma in 2, an oncocytic carcinoma in 1, and a well-differentiated follicular tumour, coded as a carcinoma by the centre, in 1. The median diameter of the largest focus was 5 mm (Q1–Q3 4–7): 58 carcinomas (95%) measured 10 mm or less and were pT1a, 2 were pT1b (15 and 18 mm), and 1 was pT2 (35 mm). In nine cases, the stage recorded by the centre was inconsistent with the recorded diameter, mainly pT3 for two carcinomas of 3 and 9 mm without coded extra-thyroidal extension and pT1b for carcinomas of 10 mm or less; accepting the three recorded pT1b stages not contradicted by the stored report would give 55 pT1a, 5 pT1b, and 1 pT2 carcinomas. Ten carcinomas (16%) were multifocal and seven (11%) showed minimal extra-thyroidal extension; none showed gross extension. Lymph nodes had been examined in 27 patients (median 2 nodes, range 1–8), none with metastasis (pN0); in the remaining 34, nodal status was not assessed (pNX), so that the absence of nodal metastasis is established only for the former.

With the available pathology, no synchronous DTC met the criteria that would mandate a more extensive operation under the 2025 ATA guidelines, although nodal status was unknown in 34 patients. Among the 13 carcinomas identified only upon review of the reports, the 47 mm follicular carcinoma met these criteria and the 12 microcarcinomas did not. Ten (16%) had at least one feature of potential management relevance (diameter > 1 cm in 3, minimal extra-thyroidal extension in 7, tall-cell subtype in 1, vascular invasion in 1); only one of these patients had undergone hemithyroidectomy. A further operation was performed in 6 of 61 patients with a synchronous DTC (completion thyroidectomy in 3 of 11 after hemithyroidectomy) and in 11 of 1117 NIFTP-only patients (10 of 307 after hemithyroidectomy). In the NIFTP-only group, at least one feature incompatible with the NIFTP definition—capsular invasion, angio-invasion, lymphatic invasion, tumour necrosis, or extra-thyroidal extension—was coded in 179 of 1124 patients (16%); 167 of them were among the patients whose stored report did not mention NIFTP, and they were concentrated in four centres (Supplementary Table S4). Of these, 177 were excluded from the strict-histology subset; the other 2 were among the patients found upon review of the reports to have a separate carcinoma, in whom the coded features were attributed to the carcinoma (Supplementary Table S7). In one patient with a synchronous DTC, the stored report described the lesion labelled NIFTP as having a breached capsule, which is incompatible with the definition; the patient was retained as coded and excluded from the strict-histology subset.

In patients with a synchronous DTC, the pre-operative ultrasound diameter of the dominant nodule was closer to the pathological diameter of the NIFTP than to that of the carcinoma in 49 of 57 informative cases, and closer to that of the carcinoma in 8, indicating that the nodule characterised before surgery was usually, but not always, the NIFTP. In the two patients with BRAF V600E-positive cytology and a synchronous DTC, the dominant nodule matched the NIFTP in size (12 and 9 mm, against carcinomas of 5 and 4 mm).

### 3.3. Performance of Pre-Operative Risk-Prediction Models

With 61 events and 16 candidate parameters (3.8 events per parameter), the expected shrinkage factor was 0.66. Performance in repeated nested cross-validation is reported in Table 3 and Supplementary Table S1. The weighted random forest achieved an AUROC of 0.56 (95% CI 0.47–0.65) and the elastic-net logistic regression 0.58 (0.48–0.67); the difference was −0.02 (−0.13 to 0.10; *p* = 0.75). Average precision was 0.087 and 0.077, respectively, against a no-skill reference of 0.051 (Supplementary Figure S4). The random forest was calibrated in the large (E/O 1.03), but its calibration slope of 0.45 (−0.21 to 1.11) indicated predictions more extreme than the information they carried; the elastic net, fitted with balanced weights, overestimated risk about ten-fold (E/O 9.7; Supplementary Figure S5) and retained no predictor in 16% of the training partitions (median 3 non-zero coefficients; Supplementary Table S3).

At the nested Youden threshold (median 0.060), the random forest classified on average 404 of the 1185 patients as positive, 24 with and 380 without a synchronous DTC, and 781 as negative, 37 of whom had a synchronous DTC: sensitivity 0.40, specificity 0.67, positive predictive value 0.06, negative predictive value 0.95, positive likelihood ratio 1.17, and negative likelihood ratio 0.90. The negative predictive value (0.953) was therefore not meaningfully higher than that obtained by classifying every patient as negative (0.949).

In leave-one-centre-out validation, there was no discrimination (pooled AUROC 0.31, 95% CI 0.24–0.38; random-effects summary of the centre-specific AUROC in the eight centres with sufficient events 0.48, 0.38–0.57), and calibration-in-the-large varied widely between centres, the observed-to-expected ratio ranging from 0 in the eight centres without recorded cases to more than 3 in eight others (random-effects summary of the centre-specific observed-to-expected ratios 1.37, 95% prediction interval 0.24–7.84; *I*^2^ 70%; Supplementary Table S9 and Figure S6; the expected-to-observed ratio of 1.35 in Table 3 refers instead to all held-out patients taken together). The pooled AUROC below 0.5 reflects the fact that centres without a recorded synchronous DTC were assigned higher average predicted risks than centres with many events. Within centres, the concordance of the cross-validated random-forest predictions was 0.48 (cluster-bootstrap 95% CI 0.37–0.54), indicating that the modest discrimination observed in cross-validation arose from differences between centres rather than between patients of the same centre. In temporal validation (development 2017–2020, 713 patients, 32 events; validation 2021–2023, 470 patients, 29 events), the AUROC was 0.39 (0.28–0.51).

**Figure 2:**
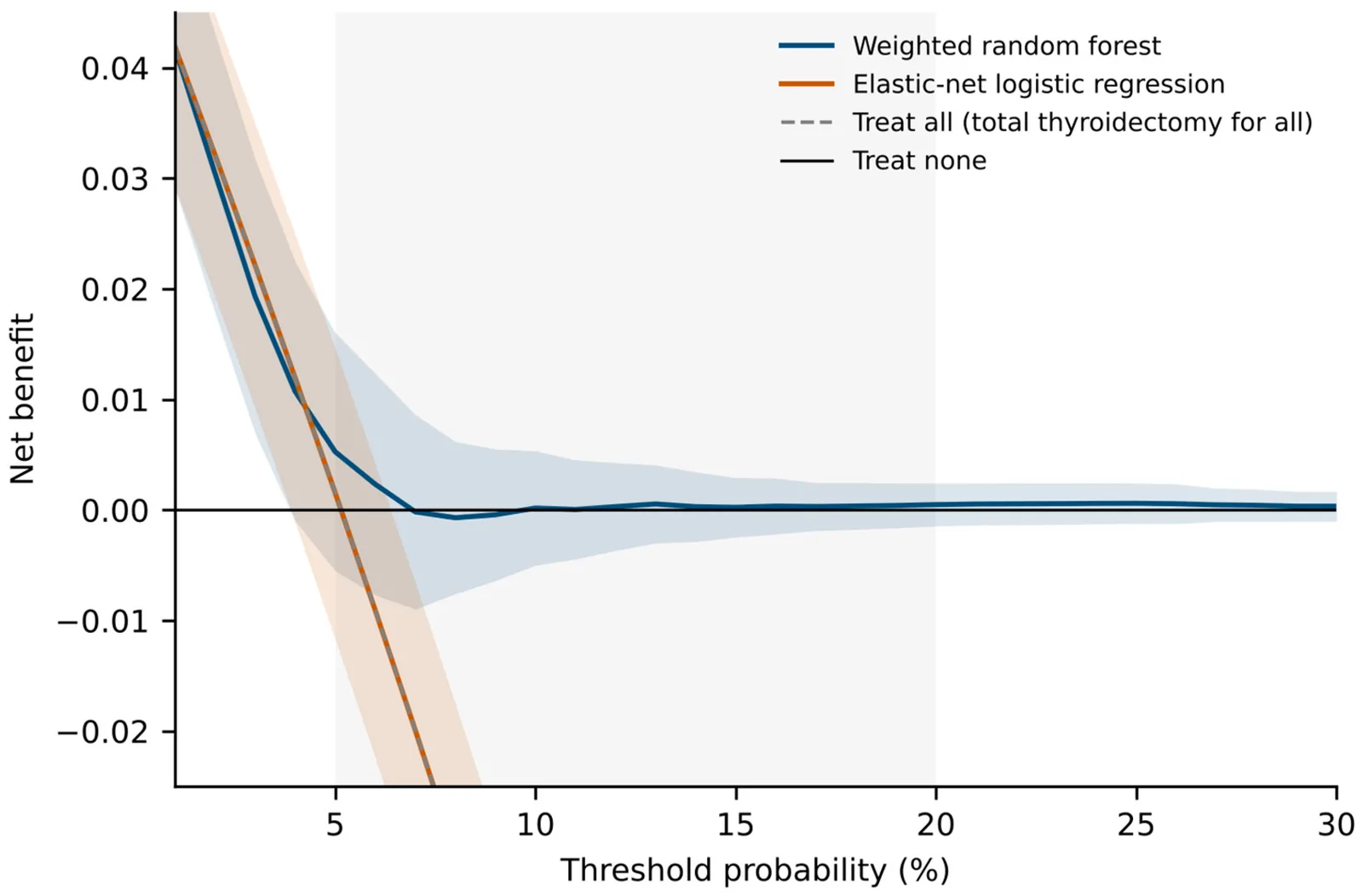
Decision-curve analysis of the cross-validated predictions of the weighted random forest and of the elastic-net logistic regression. Net benefit (with 95% CI) is plotted against the threshold probability at which total thyroidectomy rather than lobectomy would be recommended, and compared with recommending total thyroidectomy for all patients (treat all) or for none (treat none). The shaded area marks the 5–20% threshold range. The elastic-net curve coincides with treat all because the model, fitted with balanced weights, assigned predicted risks above 30% to all patients.

Decision-curve analysis (Figure 2) showed no clinically meaningful net benefit. Below a threshold probability of 5%, recommending total thyroidectomy for all patients had a net benefit equal to or greater than that of the random forest; between 5% and 20%, the net benefit of the random forest did not differ from zero (at 10%: 0.0002, 95% CI −0.0050 to 0.0053). Expressed per 1000 patients, at a 10% threshold the random forest would recommend total thyroidectomy in 50 patients, anticipating 5 of the 51 synchronous DTCs expected among 1000 patients, while 46 would still be found only at definitive histology. The elastic net assigned predicted risks above 30% to all patients, so that its decision curve coincided with that of treating all.

### 3.4. Contribution of Individual Predictors

**Figure 3:**
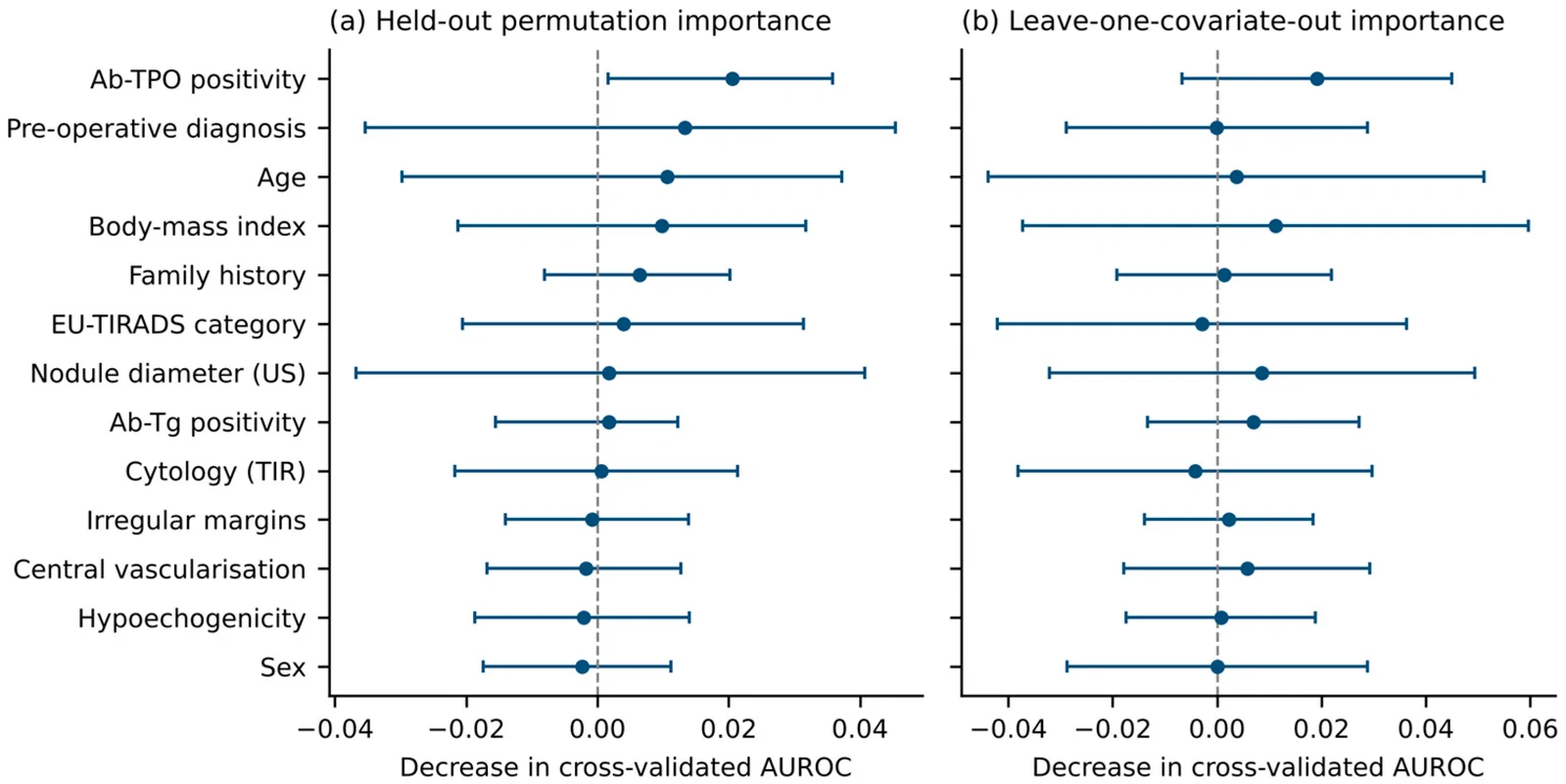
Contribution of the 13 pre-operative predictors to the weighted random forest. (**a**) Held-out permutation importance: decrease in the cross-validated AUROC when the predictor is permuted in the held-out fold (mean and 2.5th–97.5th percentiles across 20 repeats). (**b**) Leave-one-covariate-out importance: decrease in the cross-validated AUROC when the complete pipeline is refitted without the predictor (mean and 95% CI pooled across 5 repeats). Values close to zero indicate no contribution. Numerical values are reported in Supplementary Table S2.

Predictors were moderately correlated (Supplementary Figure S2), the strongest correlations being between Ab-Tg and Ab-TPO positivity (ρ = 0.74), between cytology category and pre-operative diagnosis (0.60), and between EU-TIRADS and cytology (0.46). Held-out permutation importance was small for all predictors (Figure 3 and Supplementary Table S2): only Ab-TPOs had a 95% interval excluding zero (decrease in AUROC 0.021, 0.002–0.036), and no predictor ranked among the three most important in more than 65% of the repeats. Leave-one-covariate-out importance was likewise small, with all 95% CIs including zero; the largest decreases in the AUROC were observed for Ab-TPOs (0.019, −0.007 to 0.045), BMI (0.011, −0.037 to 0.060), and nodule diameter (0.009, −0.032 to 0.049). The randomForestSRC permutation importance ranked the pre-operative diagnosis, cytology, and EU-TIRADS first, a ranking that differed both from the held-out estimates and from that of the original submission.

Partial-dependence functions (Supplementary Figure S3) showed small, non-monotonic changes in predicted risk: from about 4.5% to 7.3% across the observed range of BMI, with higher values at both extremes; from 4.8% to 7.7% across nodule diameter, with higher values for nodules of 2 mm and of 50 mm or more; and higher values above 65 years of age. The association with Ab-TPOs was inverse, Ab-TPO positivity being recorded in 23% of patients with and in 42% of those without a synchronous DTC (Table 1).

### 3.5. Sensitivity Analyses and Reconciliation with the Original Analysis

Most sensitivity analyses gave results close to those of the primary analysis (Supplementary Table S8). The AUROC of the random forest was 0.57 with the native randomForestSRC imputation nested within training partitions, 0.58 when the outcome was included in the imputation of the training data, 0.53 (0.45–0.61) with the reduced eight-predictor model, 0.57 (0.49–0.65) without class weighting, 0.54 (0.42–0.65) in patients who underwent total thyroidectomy, and 0.53 (0.44–0.62) in the 14 centres with at least one recorded case; with the adjudicated outcome (74 events), it was 0.62 (0.54–0.69), and that of the elastic net 0.65 (0.57–0.72). The within-centre concordance with the adjudicated outcome was, however, 0.48 (cluster-bootstrap 95% CI 0.41–0.55): the higher AUROC reflected differences between centres—in particular, the higher prevalence—after review of the reports, in the centre that contributed 12 of the 13 additional cases (21 of its 70 patients). In the strict-histology subset (680 patients, 66 events after review of the reports), the AUROC was 0.70 (0.62–0.78) for the random forest and 0.67 (0.57–0.75) for the elastic net, with modest discrimination within centres (within-centre concordance 0.59, 0.52–0.75; random-effects summary of the centre-specific leave-one-centre-out AUROC 0.62, 0.52–0.71, in 7 centres), but the predicted risks did not transport to new centres (pooled leave-one-centre-out AUROC 0.44, 0.37–0.51). Against the restricted outcome of a synchronous DTC of potential management relevance (10 events), the AUROC of the cross-validated random-forest predictions was 0.55 (0.34–0.75).

The clearest departure from the primary result was the missing-indicator model, which reached an AUROC of 0.75 (0.69–0.81). When missingness was allowed to act as a predictor, the pattern of recorded and unrecorded variables—strongly centre-dependent and, within centres, more complete in patients with a synchronous DTC—was, by itself, sufficient to discriminate: the apparent AUROC of the missingness indicators alone was 0.79, and that of centre membership alone 0.89. The analysis of the original submission was reproduced (out-of-bag AUROC 0.77 in the 1185 eligible patients; 0.76 in the submission). Modifying its elements one at a time showed that the estimate depended on the combination of calcitonin, the coding of the other predictors, and outcome-informed imputation. Calcitonin had been observed as 0 in 99% of the patients with a measurement but imputed with non-zero values in all those without one, and therefore identified the patients whose calcitonin had not been recorded—more often those without a synchronous DTC. With the corrected coding, the out-of-bag AUROC was 0.77 with calcitonin, 0.61 without it, and 0.53 when, in addition, imputation did not use the outcome; nesting the original pipeline within cross-validation alone reduced it to 0.71–0.73 (Supplementary Section S3 and Table S10).

## 4. Discussion

In this multicentre cohort of 1185 patients with histologically confirmed NIFTP, a synchronous differentiated thyroid carcinoma was present in 5.1% of index specimens. The carcinomas were almost invariably papillary microcarcinomas: 95% measured 10 mm or less, none had nodal metastasis among the 27 patients in whom nodes had been examined, none had gross extra-thyroidal extension, and, on the available pathology, none would have required a more extensive operation under the 2025 ATA guidelines. Review of the stored reports identified 13 further carcinomas that the centres had not coded, 12 of them microcarcinomas of 4 mm or less, which raised the prevalence to 6.2%. The figure sits at the lower end of the range reported by institutional series—from roughly 5% to more than 40%, depending on the completeness of gland sampling and on whether incidental microcarcinomas and multifocal NIFTP are counted [32,34,35,37,40]—and our data illustrate why such estimates vary: recorded prevalence ranged from none to more than 30% across centres, half of the cohort came from centres that recorded no synchronous DTC, and detection was somewhat higher after total thyroidectomy than after hemithyroidectomy (5.8% versus 3.4%, a difference compatible with chance), as expected when only resected tissue can be examined. The pooled 5.1% should therefore be read as an average over heterogeneous ascertainment rather than as a precise estimate of biological co-occurrence; restricting the analysis to centres with at least one recorded case (10.3%) or to the strict-histology subset (9.7% after review of the reports) gave higher figures. Consistent with the literature, adverse pathological features of the carcinomas were rare [33,36].

Earlier series reported considerably higher NIFTP frequencies, particularly in some European and North American centres, whereas subsequent studies have shown a progressive reduction in reported prevalence and a persistent East–West gradient [14,95]. This decline most plausibly reflects the adoption of stricter histopathological criteria, more extensive tumour sampling, and the exclusion of borderline lesions initially placed within the NIFTP spectrum, rather than a true change in disease biology alone [3,10,12]. It is a useful reminder that prevalence estimates for NIFTP—and, by extension, for synchronous DTC in NIFTP—are strongly dependent on pathological practice.

The clinical implications of the reclassification are substantial. NIFTP carries an excellent prognosis, with negligible risk of recurrence or metastasis when strict criteria are applied [35,40,96,97,98]. Recognising NIFTP as a non-malignant neoplasm therefore permits more conservative strategies, reducing the need for total thyroidectomy, lymph-node dissection, and adjuvant radioactive iodine [99,100,101], with demonstrable cost-effectiveness [4]. In our registry, follow-up information was too incomplete to assess recurrence, and features incompatible with the NIFTP definition were coded in a minority of the NIFTP-only records, a point discussed below among the limitations.

The difficulty is that this de-escalation is planned before the diagnosis is known. Because NIFTP cannot be diagnosed reliably on cytology, on ultrasound, or even on frozen section [17,19,20,102], the surgeon choosing between lobectomy and total thyroidectomy is effectively operating on an indeterminate nodule, unaware both of the NIFTP itself and of any second lesion elsewhere in the gland. In our cohort, completion thyroidectomy after hemithyroidectomy was performed in 3 of 11 patients with a synchronous DTC and in 10 of 307 without; the former reflects the fact that the discovery of a carcinoma is itself the indication for completion surgery, so that the association is definitional rather than predictive. Completion thyroidectomy carries a non-trivial additional risk of recurrent-laryngeal-nerve injury and permanent hypoparathyroidism, so avoiding it has real clinical value, yet the counterfactual—a routinely more extensive first operation—would expose the 95% of patients without a synchronous DTC to exactly the overtreatment the NIFTP reclassification was designed to prevent. A pre-operative risk estimate could inform this trade-off only if it discriminated well and if the carcinomas it anticipated were ones that would change management; neither condition was met in our data.

The individual pre-operative variables did not differ between groups after adjustment for multiplicity, and their combination did not discriminate either. In repeated cross-validation in which every data-driven step was nested, neither a weighted random forest nor a penalised logistic regression achieved useful discrimination (AUROC 0.56 and 0.58), the small signal observed was explained by differences between centres, and discrimination was absent across centres and over time; decision-curve analysis accordingly showed no net benefit over default strategies. These results supersede those of the original submission, in which an AUROC of 0.76 had been reported. The re-analysis identified the sources of that estimate (Supplementary Section S3): imputation informed by the outcome on the full dataset, evaluation and threshold selection without nesting and, above all, the pattern of missing data, which the on-the-fly imputation turned into a predictor. Calcitonin, observed as 0 in almost all measured patients but imputed with non-zero values, effectively flagged the patients in whom it had not been recorded, and missingness differed markedly between centres and, within centres, between patients with and without a synchronous DTC. In a retrospective registry, a variable that is more often recorded in patients later found to have a carcinoma reflects the process of data collection rather than pre-operative information, and a model exploiting it would not transport to new patients; consistently, allowing missingness to enter the model as a predictor reproduced an AUROC of 0.75. Some discrimination was observed only in the strict-histology subset (AUROC 0.70, within-centre concordance 0.59), and the predicted risks did not transport to new centres (pooled leave-one-centre-out AUROC 0.44); the higher AUROC obtained with the adjudicated outcome (0.62) was again explained by differences between centres. Whether routinely collected pre-operative variables carry a useful signal in a centrally reviewed cohort remains an open question.

No individual predictor carried a stable signal. Importance rankings changed with the method used; on held-out data, the intervals were compatible with no contribution for every predictor except Ab-TPOs in the permutation analysis and for every predictor in the leave-one-covariate-out analysis, whereas the randomForestSRC importance, computed within the training data, was larger for the pre-operative diagnosis, cytology, and EU-TIRADS; partial-dependence functions were flat or non-monotonic. Ab-TPO positivity was, if anything, less frequent in patients with a synchronous DTC—the opposite of the association between thyroid autoimmunity and papillary carcinoma reported in some series—and it was recorded in fewer than one third of the patients. Associations described by permutation importance or partial dependence characterise the fitted model, not the epidemiology of the disease, and with 61 events and centre-dependent recording we do not regard any of them as evidence of a risk factor [90,92].

Two methodological points have general relevance. First, inverse-frequency class weighting did not improve the discrimination of the random forest (AUROC 0.56 with and 0.57 without weighting) and, in the logistic model, produced a ten-fold overestimation of risk, while without weighting, the elastic net retained no predictor in any training partition; the same sensitivity–specificity trade-off can be obtained from an unweighted model by moving the threshold, without distorting predicted probabilities [53]. Second, out-of-bag predictions protect against optimism in tree growing but not against optimism introduced by steps performed on the full dataset—recoding, imputation with the outcome, or choice of a threshold on the same predictions used to report performance [75,76,103]; nesting the complete pipeline and assessing performance across centres are therefore necessary in multicentre data with few events [55,56,87].

Calibration is an essential complement to discrimination [81,82,104]. The calibration slope of 0.45 of the random forest indicates that its predictions spread more widely than the data justify, and centre-specific calibration-in-the-large varied widely; internally estimated calibration cannot, in any case, establish calibration in other settings. Likewise, decision-curve analysis estimates net benefit under the stated assumptions about harms and benefits and does not measure the effect of using a model on patient outcomes, which would require a prospective impact study [83,84]. The benefit assumed here—avoiding completion surgery—applies only to carcinomas for which completion would be indicated, which were rare in this cohort, so that the analysis, if anything, favours the model.

The intended use of a model of this kind deserves explicit statement. Because NIFTP is diagnosed only on the surgical specimen, a model developed in patients with NIFTP estimates the probability of a synchronous DTC conditional on a diagnosis that is unknown when the extent of surgery is chosen; the patients to whom it could be applied prospectively—those with a nodule whose histology will prove to be NIFTP—cannot be identified at that time. The only setting in which the conditioning information is available is after a lobectomy has shown NIFTP, when the question is whether the contralateral lobe may harbour a carcinoma; in that setting, too, the low frequency and low risk of the carcinomas we observed argue against completion surgery on the basis of pre-operative features.

From a management standpoint, our findings support a graded approach that does not depend on a pre-operative risk estimate. For isolated NIFTP, lobectomy is adequate and, in selected low-risk lesions, follow-up alone may be defensible given the indolent behaviour of the entity [8,9,41]. When a synchronous DTC is present, treatment and surveillance should follow ATA risk stratification for differentiated thyroid carcinoma and be driven by the size, stage, and histotype of that carcinoma, not by the NIFTP [7,8]. Since the synchronous DTCs in our cohort were overwhelmingly intrathyroidal microcarcinomas without documented nodal involvement, completion thyroidectomy will seldom be indicated after lobectomy, and surveillance—well established for low-risk papillary microcarcinoma—should be considered [42,43,44]. Our data do not support selecting patients for total thyroidectomy on the basis of pre-operative clinical, ultrasonographic, cytological, or biochemical features.

This study has several strengths. It is, to our knowledge, among the largest multicentre series of NIFTP with a second histological review; the synchronous DTCs were reconciled case by case from the registry fields and the stored reports; and the prediction analysis was designed to expose optimism: all data-driven steps were nested within repeated cross-validation, transportability was assessed across centres and over time, missing-data assumptions were varied, the random forest was benchmarked against a penalised logistic regression, and multiplicity in the descriptive analysis was controlled with the Benjamini–Yekutieli procedure.

Several limitations should be acknowledged. First, the design is retrospective and based on a registry compiled by the participating centres; the number of thyroidectomies from which the cohort was drawn was not recorded, so that selection at the centre level cannot be assessed, and outcome ascertainment clearly differed between centres: review of the stored reports found 13 carcinomas not coded by the centres, and the reports that did not name NIFTP could not be reviewed in the same way. Second, the NIFTP diagnosis could not be re-verified centrally. A second review was performed locally; for 41% of the patients, the stored report used pre-2016 terminology or a TNM category only, in 16% of the NIFTP-only records, the coded histological fields reported invasion, extra-thyroidal extension, or necrosis, which are incompatible with the definition, and 33 NIFTP-only patients, 31 from one centre, had BRAF V600E-positive cytology. Part of the registry may therefore include invasive encapsulated follicular-variant carcinomas or other follicular-patterned tumours rather than NIFTP [12,29,30], which is why the strict-histology subset is reported alongside the full cohort. Third, nodal status was assessed in fewer than half of the patients with a synchronous DTC, and the absence of nodal metastasis cannot be extended to the others. Fourth, pre-operative ultrasound, cytology, and molecular results were recorded for the dominant nodule without explicit linkage to the lesion eventually diagnosed; the diameters suggest that this was the NIFTP in most cases but the carcinoma in some, and ultrasonographic features were assessed locally without interobserver standardisation across centres. Fifth, missing data were extensive and their pattern depended on centre and outcome; the registry does not record why a value is missing, so that tests not performed cannot be distinguished from results not retrieved, and no imputation method can recover information that was not collected, and the poor discrimination we report applies to the variables as they are recorded in routine practice [72,105]. Sixth, with 61 events, the study was too small to develop a stable multivariable model, and a sample several times larger would be needed even for a model of modest complexity [64,66]; the absence of discrimination should, however, be read together with the univariate comparisons and the centre-aware analyses, which point in the same direction. No independent external cohort was available, and internal–external and temporal validation were used as the closest approximation [55]. Seventh, follow-up was too incomplete to study recurrence, and the cohort is predominantly Italian, whereas NIFTP frequency and the prevalence of synchronous DTC vary geographically [14,38].

Future work should rely on prospective, standardised data collection with explicit linkage of pre-operative findings to the lesions eventually diagnosed, central pathological review, and complete recording of nodal assessment. Molecular markers such as BRAF V600E and RAS mutations, and emerging proteomic signatures, may be more informative than routine clinical variables [25,26,106], and artificial-intelligence-assisted pathology may improve the reproducibility of the distinction between NIFTP and invasive follicular-variant papillary carcinoma [10,107]. Any new model should be developed in the population in which it would be used—patients with indeterminate nodules scheduled for surgery—and validated externally before clinical use [54,57].

## 5. Conclusions

Approximately one in twenty NIFTP-containing surgical specimens harboured a synchronous differentiated thyroid carcinoma, usually an intrathyroidal papillary microcarcinoma without nodal metastasis where nodes were examined, which would not by itself require more extensive surgery under current guidelines; recorded frequencies vary widely between centres. Routinely available pre-operative clinical, ultrasonographic, cytological, and biochemical variables, whether combined by a random forest or by a penalised logistic regression, did not identify these patients once optimism was controlled and differences between centres were taken into account. Management of a synchronous DTC should be guided by its pathological features, and the extent of the initial operation for nodules in the NIFTP spectrum cannot at present be tailored on the basis of such variables. Meticulous histopathological sampling, adherence to the WHO 2022 and ATA 2025 criteria, and standardised prospective data collection are prerequisites for any future attempt at pre-operative risk stratification.

## Figures and Tables

**Table 1:** Pre-operative clinical, ultrasonographic, cytological, and biochemical characteristics of the cohort, stratified by the presence of a synchronous differentiated thyroid carcinoma (DTC).

Variable	NIFTP Only (*n* = 1124)	NIFTP + DTC (*n* = 61)	*q*-Value
**Demographics**			
Female sex, *n*/*N* (%)	832/1122 (74)	44/61 (72)	1.00
Age, years, median (Q1–Q3)	52 (41–62)	55 (46–66)	1.00
Body-mass index, kg/m^2^, median (Q1–Q3)	25.0 (22.0–28.1)	25.4 (22.0–30.8)	1.00
Family history of thyroid disease, *n*/*N* (%)			1.00
None	389/531 (73)	29/32 (91)	
Benign thyroid disease	120/531 (23)	3/32 (9)	
Thyroid malignancy	22/531 (4)	0/32 (0)	
Pre-operative clinical diagnosis, *n*/*N* (%)			1.00
Indeterminate nodule	465/1123 (41)	19/61 (31)	
Goitre/multinodular goitre	306/1123 (27)	19/61 (31)	
Benign nodule	45/1123 (4)	4/61 (7)	
Suspicious for malignancy	307/1123 (27)	19/61 (31)	
**Ultrasonography (dominant nodule)**			
EU-TIRADS category, *n*/*N* (%)			1.00
1	22/991 (2)	4/57 (7)	
2	257/991 (26)	13/57 (23)	
3	340/991 (34)	16/57 (28)	
4 (including 4A/4B)	253/991 (26)	17/57 (30)	
5	119/991 (12)	7/57 (12)	
Echogenicity, *n*/*N* (%)			1.00
Hypoechoic	436/795 (55)	29/43 (67)	
Isoechoic	185/795 (23)	4/43 (9)	
Hyperechoic	90/795 (11)	5/43 (12)	
Anechoic	84/795 (11)	5/43 (12)	
Vascularisation, *n*/*N* (%)			0.95
Peripheral	281/669 (42)	22/40 (55)	
Central and peripheral	386/669 (58)	17/40 (42)	
Absent	2/669 (<1)	1/40 (2)	
Sonographic margins, *n*/*N* (%)			1.00
Regular	521/677 (77)	34/44 (77)	
Irregular	156/677 (23)	10/44 (23)	
Nodule diameter, mm, median (Q1–Q3)	20 (10–35)	20 (10–40)	1.00
**Cytology**			
TIR (Bethesda) category, *n*/*N* (%)			1.00
TIR 2 (Bethesda II)	165/962 (17)	7/56 (12)	
TIR 3a (Bethesda III)	207/962 (22)	11/56 (20)	
TIR 3b (Bethesda IV)	360/962 (37)	22/56 (39)	
TIR 4 (Bethesda V)	160/962 (17)	12/56 (21)	
TIR 5 (Bethesda VI)	70/962 (7)	4/56 (7)	
**Pre-operative molecular testing (FNAC)**			
Molecular profile, *n*/*N* (%)			1.00
Wild type	534/589 (91)	24/27 (89)	
BRAF V600E	33/589 (6)	2/27 (7)	
HRAS/KRAS/NRAS	22/589 (4)	1/27 (4)	
**Pre-operative biochemistry, *n*/*N* (%)**			
Calcitonin elevated	4/349 (1)	0/46 (0)	1.00
Ab-Tgs, positive	161/358 (45)	12/37 (32)	1.00
Ab-TPOs, positive	140/334 (42)	8/35 (23)	0.95
**Surgery**			
Initial operation, *n*/*N* (%)			1.00
Hemithyroidectomy	310/1123 (28)	11/61 (18)	
Total thyroidectomy	813/1123 (72)	50/61 (82)	

*n*: number with the characteristic; *N*: number with available data. Q1–Q3: 25th–75th percentile. *q*-values are *p*-values from Fisher’s exact test (categorical variables) or the Wilcoxon rank-sum test (continuous variables) after Benjamini–Yekutieli adjustment across the 16 comparisons in the table. Ultrasound, cytology, and molecular results refer to the dominant nodule that prompted surgery. The case-report-form code ‘not determined’ for echogenicity, vascularisation, and margins is treated as missing; calcitonin and antibodies were recorded as elevated/positive or negative according to local laboratory reference ranges. Missing data by variable, outcome, and centre are reported in Supplementary Table S6. Ab-Tgs, anti-thyroglobulin antibodies; Ab-TPOs, anti-thyroperoxidase antibodies; DTC, differentiated thyroid carcinoma; EU-TIRADS, European Thyroid Imaging Reporting and Data System; FNAC, fine-needle-aspiration cytology; NIFTP, non-invasive follicular thyroid neoplasm with papillary-like nuclear features; TIR, Italian Society of Endocrine Pathology cytology classification.

**Table 2:** Pathological characteristics of the index lesion and of the synchronous differentiated thyroid carcinoma (DTC), stratified by group.

Variable	NIFTP Only (*n* = 1124)	NIFTP + DTC (*n* = 61)
**Index NIFTP lesion**		
Pathological diameter, mm, median (Q1–Q3)	17 (8–30)	20 (11–39)
Associated chronic thyroiditis, *n*/*N* (%)	283/1118 (25)	17/61 (28)
**Microscopic features coded at specimen level, *n*/*N* (%)**		
Any of the features below	179/1124 (16) ^a^	12/61 (20) ^b^
Capsular invasion	76/1123 (7)	5/61 (8)
Angio-invasion	56/1122 (5)	1/61 (2)
Lymphatic invasion	69/1116 (6)	0/60 (0)
Tumour necrosis	24/1115 (2)	1/61 (2)
Extra-thyroidal extension	76/1123 (7)	7/61 (11)
**Synchronous DTC**		
Histotype, *n*/*N* (%)	—	
Papillary carcinoma	—	57/61 (93)
Classic subtype	—	6/61 (10)
Follicular subtype	—	3/61 (5)
Tall-cell subtype	—	1/61 (2)
Subtype not specified	—	47/61 (77)
Follicular carcinoma	—	2/61 (3)
Oncocytic carcinoma	—	1/61 (2)
Well-differentiated follicular tumour	—	1/61 (2)
Diameter of largest focus, mm, median (Q1–Q3)	—	5 (4–7)
10 mm or less, *n*/*N* (%)	—	58/61 (95)
Multifocal, *n*/*N* (%)	—	10/61 (16)
Extra-thyroidal extension, *n*/*N* (%)	—	
Minimal (microscopic)	—	7/61 (11)
Gross	—	0/61 (0)
pT, AJCC 8th edition (largest focus), *n*/*N* (%) ^c^	—	
pT1a	—	58/61 (95)
pT1b	—	2/61 (3)
pT2	—	1/61 (2)
pT3–pT4	—	0/61 (0)
pN, *n*/*N* (%)	—	
pN0 (≥1 node examined, none metastatic)	—	27/61 (44)
pNX (no node examined)	—	34/61 (56)
pN1	—	0/61 (0)
Features mandating more extensive surgery ^d^	—	0/61 (0)
Features of potential management relevance ^d^	—	10/61 (16)
**Further surgery, *n*/*N* (%)**		
Completion or re-operation, all patients	11/1117 (1)	6/61 (10)
Completion after hemithyroidectomy	10/307 (3)	3/11 (27)
Re-operation after total thyroidectomy	1/809 (<1)	3/50 (6)

*n*: number with the characteristic; *N*: number with available data. Q1–Q3: 25th–75th percentile. ‘—’ indicates that the variable is not defined in that group. ^a^ Features coded at specimen level; in specimens coded as NIFTP-only, they refer only to the index lesion, unless a separate carcinoma was present but not coded (2 patients on review of the reports), and are incompatible with the NIFTP definition. A total of 167 of the 179 patients belonged to the group whose stored report did not mention NIFTP; 177 were excluded from the strict-histology subset. ^b^ In specimens with a synchronous DTC, attributed to the carcinoma after case-level review (Supplementary Table S7); in one patient, the stored report attributed a breached capsule to the lesion labelled NIFTP. ^c^ Size-derived AJCC eighth-edition category of the largest focus; the stage recorded by the centre was discordant in nine cases, and accepting the three recorded pT1b stages not contradicted by the stored report would give 55 pT1a, 5 pT1b, and 1 pT2. ^d^ Definitions in Section 2.2; assessed on the available pathology (nodal status unknown in the 34 pNX patients). Because the identification of a synchronous DTC is itself the indication for completion surgery, the difference in further surgery is expected by design. AJCC, American Joint Committee on Cancer; ATA, American Thyroid Association; DTC, differentiated thyroid carcinoma; NIFTP, non-invasive follicular thyroid neoplasm with papillary-like nuclear features.

**Table 3:** Performance of the pre-operative risk-prediction models for synchronous differentiated thyroid carcinoma (estimates with 95% CI).

Measure	Random Forest, Nested CV	Elastic Net, Nested CV	Random Forest, Leave-One-Centre-Out	Random Forest, Temporal ^d^
**Discrimination and calibration**				
AUROC	0.56 (0.47 to 0.65)	0.58 (0.48 to 0.67)	0.31 (0.24 to 0.38)	0.39 (0.28 to 0.51)
Average precision ^a^	0.087 (0.045 to 0.162)	0.077 (0.043 to 0.137)	0.035 (0.026 to 0.046)	0.063 (0.031 to 0.124)
Brier score	0.049 (0.038 to 0.060)	0.247 (0.245 to 0.250)	0.052 (0.041 to 0.063)	0.060 (0.040 to 0.080)
Calibration-in-the-large	−0.03 (−0.29 to 0.23)	−2.90 (−3.16 to −2.65)	−0.33 (−0.60 to −0.06)	0.31 (−0.11 to 0.72)
Calibration slope	0.45 (−0.21 to 1.11)	5.86 (−4.30 to 16.03)	−1.48 (−1.99 to −0.97)	−0.37 (−0.82 to 0.09)
Expected/observed ratio	1.03 (0.80 to 1.33)	9.66 (7.50 to 12.45)	1.35 (1.04 to 1.76)	0.76 (0.50 to 1.14)
**Classification at the nested Youden threshold**				
Threshold (median)	0.060	0.482	0.059	0.033
TPs/FPs/FNs/TNs ^b^	24/380/37/744	47/790/14/334	29/529/32/595	11/254/18/187
Sensitivity	0.40 (0.21 to 0.62)	0.76 (n.e.)	0.48 (0.36 to 0.60)	0.38 (0.22 to 0.57)
Specificity	0.67 (0.52 to 0.78)	0.27 (0.05 to 0.70)	0.53 (0.50 to 0.56)	0.42 (0.38 to 0.47)
Positive predictive value	0.060 (0.037 to 0.095)	0.056 (0.041 to 0.076)	0.052 (0.036 to 0.075)	0.042 (0.024 to 0.072)
Negative predictive value	0.953 (0.934 to 0.967)	0.962 (n.e.)	0.949 (0.930 to 0.963)	0.912 (0.865 to 0.944)
Positive likelihood ratio	1.17 (0.77 to 1.79)	1.09 (0.88 to 1.36)	1.01 (0.77 to 1.32)	0.66 (0.41 to 1.07)
Negative likelihood ratio	0.90 (0.69 to 1.18)	0.73 (n.e.)	0.99 (0.78 to 1.27)	1.46 (1.07 to 1.99)
**Clinical utility**				
Net benefit at 10% threshold ^c^	0.0002 (−0.0050 to 0.0053)	−0.0539 (−0.0678 to −0.0400)	−0.0092 (−0.0134 to −0.0049)	−0.0021 (−0.0097 to 0.0054)

Nested CV: 20-times repeated five-fold cross-validation with imputation, tuning, class weighting, and threshold selection nested within training partitions (1185 patients, 61 events); estimates pooled across repeats with Rubin’s rules. Leave-one-centre-out: each of the 22 centres held out in turn. Temporal: development on patients operated on in 2017–2020 (713 patients, 32 events) and validation on those operated on in 2021–2023 (470 patients, 29 events). ^a^ No-skill reference equal to the outcome prevalence (0.051; 0.062 in the temporal validation set). ^b^ Mean counts across repeats; proportions are pooled on the logit scale and may differ slightly from those computed from the mean counts. ^c^ Net benefit of treating all patients at the 10% threshold: −0.054 (−0.043 in the temporal validation set). ^d^ Single development–validation split. n.e., not estimable (sensitivity equal to 1 in some repeats; the mean across repeats is shown). AUROC, area under the receiver operating characteristic curve; CV, cross-validation; FNs, false negatives; FPs, false positives; TNs, true negatives; TPs, true positives.

## Data Availability

The analysis code of the revised analysis and the de-identified data underlying the descriptive analyses are available from the corresponding author upon reasonable request, subject to the data-sharing agreement of the participating centres and to compliance with the EU General Data Protection Regulation.

## Supplementary Materials

The following supporting information can be downloaded at https://www.mdpi.com/article/10.3390/cancers18213505/s1. Section S1: Statistical Methods; Section S2: Reproducibility; Section S3: Reconciliation with the Analysis of the Original Submission; Table S1: Performance of the pre-operative risk-prediction models (estimates with 95% CI); Table S2: Contribution of individual predictors to the weighted random forest; Table S3: Hyperparameter configuration and tuning of the weighted random forest; Table S4: Centre-level recruitment, outcome prevalence and data completeness; Table S5: Predictor dictionary; Table S6: Missing data by predictor, outcome and centre; Table S7: Case-level reconciliation of the synchronous DTCs: (A) the 61 coded by the centres; (B) 13 carcinomas described in the stored reports of patients coded as NIFTP-only; Table S8: Sensitivity analyses; Table S9: Leave-one-centre-out validation of the weighted random forest; Table S10: Reconciliation with the analysis of the original submission (1185 eligible patients); Figure S1: Percentage of missing values by predictor and centre. Centre labels give the number of patients and of synchronous DTCs; Figure S2: Spearman correlation matrix of the pre-operative predictors (pairwise complete observations; binary predictors coded 0/1; the pre-operative diagnosis represented by indicators of suspicious nodule and goitre); Figure S3: Partial-dependence functions of the weighted random forest for body-mass index, nodule diameter, age, EU-TIRADS category and cytology category: median predicted risk (line) and 2.5th–97.5th percentiles (band) across the 100 training partitions of the nested cross-validation; Figure S4: Receiver operating characteristic (a) and precision–recall (b) curves of the out-of-fold predictions of the weighted random forest and of the elastic-net logistic regression in each of the 20 repeats of nested cross-validation. The dashed line in (b) is the no-skill reference (outcome prevalence); Figure S5: Calibration of the cross-validated predictions (mean of 20 repeats per patient): observed proportion by decile of predicted risk with 95% CI and loess smoother. Note the different horizontal scales: the elastic net, fitted with balanced weights, predicts risks close to 0.5 for all patients; Figure S6: Leave-one-centre-out validation: centre-specific AUROC (left; centres with at least three patients with and without the outcome) and observed-to-expected ratio of synchronous DTCs (right; log scale, 0.5 added to observed and expected counts). The vertical line on the left marks the random-effects summary AUROC.
