# Supplementary material for: Synchronous Differentiated Thyroid Carcinoma in Non-Invasive Follicular Thyroid Neoplasm with Papillary-like Nuclear Features (NIFTP): Prevalence, Pathological Profile, and Limits of Pre-Operative Risk Prediction in a Multicentre Cohort
Source: Cancers (Basel). 2026 Oct 30;18(21):3505. doi: 10.3390/cancers18213505 (PMC13761702; doi:10.3390/cancers18213505)
Supplement: Supplementary file 1 [file cancers-18-03505-s001.zip › cancers-4575217-supplementary.pdf]

Supplementary Materials

Synchronous Differentiated Thyroid Carcinoma in Non-Invasive Follicular Thyroid Neoplasm with Papillary-like Nuclear Features (NIFTP): Prevalence, Pathological Profile, and Limits of Pre-Operative Risk Prediction in a Multicentre Cohort

Chiara Dobrinja, Manuela Mastronardi, Stella Bernardi, Elena Bonati, Marco Bononi, Pietro Giorgio Calò, Gian Luigi Canu, Federico Cappellacci, Maria Grazia Chiofalo, Loredana De Pasquale, Carmela De Crea, Marco Stefano Demarchi, Paolo Del Rio, Giacomo Di Filippo, Giovanni Docimo, Daniele Gasparini, Rita Gervasi, Giuseppa Graceffa, Angela Gurrado, Maurizio Iacobone, Eduardo Mercelo Iervolino, Nadia Innaro, Rita Laforgia, Giovanni Lazzari, Petra Makovac, Gabriele Materazzi, Fabio Medas, Eleonora Morelli, Barbara Mullineris, Piermarco Papini, Francesco Pennestrì, Angela Pezzolla, Marco Raffaelli, Esther Diana Rossi, Leonardo Rossi, Gregorio Scerrino, Luca Sessa, Mario Testini, Emanuela Traini, Nicolò de Manzini and Dario Gregori

Contents. Section S1. Statistical methods. Section S2. Reproducibility. Section S3. Reconciliation with the analysis of the original submission. Tables S1–S10. Figures S1–S6.

Section S1. Statistical Methods

S1.1. Cohort, outcome and predictors

The analysed cohort comprised the 1185 registry patients aged 18 years or older with NIFTP confirmed at second histological review and operated on between 1 January 2017 and 31 December 2023 in 22 centres (main text, Figure 1). Each patient contributed the specimen of the index operation. The outcome was the presence of a synchronous differentiated thyroid carcinoma (DTC) in that specimen, as coded by the centre in a dedicated binary field; 61 patients had the outcome. An adjudicated outcome, which adds the carcinomas described in the stored reports but not coded by the centres, was used in sensitivity analyses (S1.10). Fourteen pre-operative candidate predictors were prespecified (Table S5). Calcitonin was elevated in 4 of 395 patients with a measurement, none with a synchronous DTC, and was excluded from modelling as a near-constant variable; the remaining 13 predictors correspond to 16 model parameters (one each for the continuous, ordinal and binary predictors, two for family history and three for the pre-operative clinical diagnosis). Post-operative variables and intra-operative decisions were excluded to prevent look-ahead bias.

S1.2. Descriptive analysis and multiplicity

Categorical variables were compared with Fisher's exact test (Monte Carlo p-values with 10^5 replicates for tables larger than 2×2) and continuous variables with the Wilcoxon rank-sum test. The 16 p-values of Table 1 were adjusted with the Benjamini–Yekutieli procedure. Heterogeneity of prevalence across centres was assessed with Fisher's exact test and described with a random-intercept logistic model (lme4), from which the median centre prevalence and the 95% prediction interval for a new centre were derived. Temporal trends were assessed by adding year of surgery as a linear term.

S1.3. Missing data

Missing predictor values were imputed by multiple imputation by chained equations (mice 3.16.0) with five imputations and ten iterations: predictive mean matching for continuous and ordinal variables (age, BMI, nodule diameter, EU-TIRADS, cytology), logistic regression for binary variables (sex, echogenicity, vascularisation, margins, Ab-Tg, Ab-TPO) and polytomous regression for nominal variables (family history, pre-operative diagnosis); every predictor was used to impute every other. The outcome was excluded from the imputation model. Within each training partition, the imputation model was estimated on the training patients only (argument *ignore* of mice for the held-out patients) and used to impute both training and held-out patients, so that no information from held-out patients, and no outcome, entered the imputation of the held-out data. Each model was fitted separately to each of the five imputed training sets, and the five predicted probabilities of each held-out patient were averaged.

S1.4. Models and hyperparameter tuning

Weighted random forest. randomForestSRC 3.4.5, classification forest with AUC-based splitting and 10 random split points per candidate variable; class weights inversely proportional to the outcome frequencies in the training partition and normalised to sum to one; 1000 trees per imputed dataset. The number of candidate variables per split (2, 3, 5) and the minimum terminal-node size (3, 10, 30) were selected by maximising the out-of-bag AUROC of a 500-tree forest on the first imputed training set (Table S3). *Elastic-net logistic regression.* glmnet 4.1-8, mixing parameter $\alpha = 0.5$, observation weights balancing the two classes as in the original submission, λ selected by ten-fold cross-validation (binomial deviance) within the training partition with the one-standard-error rule; continuous and ordinal predictors entered linearly and nominal predictors as indicator variables, all standardised internally. The λ minimising deviance was examined as a sensitivity analysis (Table S1).

S1.5. Nested internal validation and pooling

Twenty repeats of outcome-stratified five-fold cross-validation were run with different random partitions; the same partitions were used for both models. For each training partition the complete pipeline—imputation, tuning, class weighting, model fitting and threshold selection—was run on the training patients only and the resulting rule was applied once to the held-out fold. Within each repeat the out-of-fold predictions of all 1185 patients (and the fold-specific thresholds) were pooled, and performance measures were computed on the pooled predictions. For each measure q, the 20 repeat-specific estimates were transformed (logit for proportions and the AUROC, log for ratios, none for the Brier score, calibration measures and net benefit), the pooled estimate is their mean, back-transformed, and its variance on the transformed scale is $T = W + (1 + 1/20)B$, where W is the mean within-repeat variance (DeLong variance for the AUROC; variance over 200 bootstrap resamples of the pooled predictions otherwise) and B the variance between repeats. When a proportion equalled 0 or 1 in some repeats, the interval was not estimable and the untransformed mean across repeats is reported. The scaled Brier score, $1 - \text{Brier}/[\pi(1 - \pi)]$, is a linear function of the Brier score and was derived from the pooled Brier score. Calibration-in-the-large was the intercept of a logistic model with the logit of the predicted probability as offset, and the calibration slope the coefficient of the logit in a logistic model without offset. The paired comparison of the two models used the DeLong test within each repeat, with the difference in AUROC pooled as above.

S1.6. Threshold selection and decision-curve analysis

Within each training partition, the classification threshold was the one maximising the Youden index (sensitivity + specificity − 1) on predictions internal to the training data: out-of-bag predictions for the random forest and inner cross-validated (pre-validated) predictions at the selected λ for the elastic net. For decision-curve analysis, a positive classification at threshold

probability p_t represents a pre-operative recommendation of total thyroidectomy rather than lobectomy. Net benefit was TP/n − FP/n × $p_t/(1 - p_t)$ and that of treating all patients $\pi - (1 - \pi) \times p_t/(1 - p_t)$, π being the prevalence; net benefit was computed on the pooled out-of-fold predictions of each repeat for p_t from 1% to 30% and pooled across repeats as above. At p_t = 10%, one true positive is worth nine false positives; the per-1000 counts reported in the main text are the mean numbers of true and false positives across repeats divided by 1185 and multiplied by 1000.

S1.7. Sample size and model complexity

The minimum sample size for model development was calculated with the criteria of Riley et al. (pmsampsize 1.1.3), assuming an outcome prevalence of 5.1%, a target shrinkage factor of 0.9 and anticipated C-statistics of 0.65, 0.70 and 0.75 (Cox–Snell R^2 0.014, 0.026 and 0.042). The expected shrinkage for the available sample (1185 patients, 61 events) was obtained by solving $n = P/[(S - 1)\ln(1 - R^2_{CS}/S)]$ for S, and the maximum number of parameters supported for S = 0.9 as $P = n(S - 1)\ln(1 - R^2_{CS}/S)$.

Anticipated C-statistic	Cox–Snell R^2	Required n (events), 10 parameters	Required n (events), 16 parameters	Expected shrinkage, 16 parameters	Maximum parameters for S = 0.9
0.65	0.014	6288 (324)	10,061 (518)	0.52	1.9
0.70	0.026	3412 (176)	5459 (282)	0.66	3.5
0.75	0.042	2083 (108)	3332 (172)	0.76	5.7

Calculated for an outcome prevalence of 5.1% and, for the last two columns, for the available 1185 patients.

S1.8. Centre-aware and temporal validation

In internal–external cross-validation each of the 22 centres was held out in turn and the complete pipeline was developed on the other 21. Pooled performance was computed on the 1185 held-out predictions. Centre-specific AUROCs (with DeLong standard errors) were computed for the 8 centres with at least three patients with and three without the outcome and summarised by random-effects meta-analysis on the logit scale (metafor, restricted maximum likelihood); observed-to-expected ratios were computed for all centres and summarised on the log scale after adding 0.5 to observed and expected counts. Within-centre discrimination was the centre-stratified concordance statistic (concordant pairs formed only within centres) of the patient-level mean of the 20 cross-validated predictions, with a 95% CI from cluster-bootstrap resamples of centres (1000 for the primary analysis, 500 for the sensitivity analyses). For temporal validation the pipeline was developed on the 713 patients operated on in 2017–2020 and applied to the 470 operated on in 2021–2023 (2 patients without a recorded date were excluded from this analysis).

S1.9. Contribution of individual predictors

Held-out permutation importance. In each fold, the values of one predictor were randomly permuted among the held-out patients (the same permutation applied to the five imputed held-out sets), the fitted forests were applied unchanged, and the predictions were averaged across imputations; the outcome was not permuted and no model was refitted. The importance is the decrease in the AUROC of the pooled out-of-fold predictions of each repeat, summarised by the mean and the 2.5th–97.5th percentiles across the 20 repeats, and by the proportion of repeats in which the predictor ranked among the three most important. *Leave-one-covariate-out (LOCO) importance.* The pipeline was refitted without each predictor in each training partition (five repeats, 500 trees per imputed dataset, tuned hyperparameters of the full model), and the decrease in cross-validated AUROC relative to the full model was pooled across repeats with the paired DeLong variance and Rubin's rules. *randomForestSRC permutation importance.* Breiman–Cutler importance with the Brier score as performance measure, obtained in each of the 100 training forests (averaged over the five imputed datasets): for each tree, the predictor is permuted among

the out-of-bag cases, and the change in error is averaged over trees; summarised by the median and the 2.5th–97.5th percentiles across the 100 training partitions. *Partial dependence.* For BMI, nodule diameter, age, EU-TIRADS and cytology, the predictor was set to each grid value for all training patients of the first imputed set and the predictions of the first forest averaged; median and 2.5th–97.5th percentile curves across the 100 training partitions are shown in Figure S3.

S1.10. Sensitivity analyses

Ten repeats of five-fold cross-validation were used for each scenario (Table S8): (i) native randomForestSRC on-the-fly imputation (three iterations) nested within training partitions, with held-out patients imputed by the training forest without the outcome; (ii) outcome included in the imputation of the training patients, held-out patients being imputed by a separate model without the outcome; (iii) missing-indicator coding, with missing values replaced by the training median or mode and an indicator of missingness added for each predictor with missing values in the training partition; (iv) a reduced prespecified model with the eight predictors with less than 30% missing values (sex, age, BMI, pre-operative diagnosis, EU-TIRADS, cytology, echogenicity, nodule diameter; 10 parameters); (v) no class weighting; (vi) patients who underwent total thyroidectomy; (vii) the 14 centres with at least one recorded synchronous DTC; (viii) the adjudicated outcome; and (ix) the strict-histology subset. *Adjudicated outcome.* The free text of the 637 stored reports of NIFTP-only patients that explicitly named NIFTP was searched for the terms carcinoma, microcarcinoma, microtumour, papillary neoplasm and their abbreviations, and every hit (43 reports) was read. Thirteen reports described a carcinoma separate from the NIFTP (Table S7, part B); these patients were counted as having a synchronous DTC (74 events); 12 of them belong to the strict-histology subset (one was excluded for BRAF V600E-positive cytology). In two of them a feature incompatible with NIFTP had been coded at specimen level and was attributed to the carcinoma. Reports that did not name NIFTP describe the index lesion in the terminology of the original diagnosis and could not be reviewed in this way. *Strict-histology subset.* Patients whose stored report explicitly named NIFTP, who had no BRAF V600E mutation on pre-operative cytology, and in whom no feature incompatible with NIFTP was reported for the index lesion: for patients without a synchronous DTC, no coded capsular invasion, angio-invasion, lymphatic invasion, tumour necrosis or extra-thyroidal extension and no statement of invasion or capsular breach in the report (statements of a lymphocytic or inflammatory infiltrate, or negated statements, were not counted); for patients with a synchronous DTC, whose coded features were attributed to the carcinoma, the statements in the report were read and excluded the patient only if they referred to the NIFTP (one report). The adjudicated outcome was used (680 patients, 66 events; 54 as coded by the centres). The cross-validated predictions of the primary analysis were also evaluated against the outcome of a synchronous DTC of potential management relevance (larger than 1 cm, minimal extra-thyroidal extension, vascular invasion or tall-cell subtype; 10 events).

Section S2. Reproducibility

The re-analysis was performed in R 4.3.3 with randomForestSRC 3.4.5, glmnet 4.1-8, mice 3.16.0, pROC 1.18.5, metafor 4.4-0, lme4 and pmsampsize 1.1.3; random seeds are fixed in the code. The analysis code and the de-identified data underlying the descriptive analyses are available from the corresponding author on reasonable request, subject to the data-sharing agreement of the participating centres and to compliance with the EU General Data Protection Regulation. The TRIPOD+AI, TRIPOD-Cluster and STROBE checklists are provided as separate files.

Section S3. Reconciliation with the Analysis of the Original Submission

The originally submitted analysis (weighted random forest with the 14 predictors in their original coding, randomForestSRC on-the-fly imputation on the full dataset, out-of-bag evaluation) was reproduced in the 1185 eligible patients, with an out-of-bag AUROC of 0.77 (0.76 in the submission). Its elements were then modified one at a time (Table S10). Recoding the 'not determined' categories as missing did not change the estimate (0.76), and nesting the original pipeline within cross-validation reduced it to 0.71–0.73. Removing calcitonin reduced the AUROC to 0.72 with the original coding; with the corrected coding the AUROC was 0.77 with calcitonin and 0.61 without it, falling to 0.53 when imputation did not use the outcome. The estimate therefore depended on the combination of calcitonin, the coding of the other predictors and outcome-informed imputation, and its optimism was further increased by the absence of nesting.

The mechanism was verified directly. Calcitonin was recorded as 0 in 99% of the patients with a measurement, whereas the on-the-fly imputation replaced every missing value with a non-zero value (median 0.44, range 0.005–2.1), and the same occurred for the antibodies, recorded as 0 or 1 and imputed with non-integer values. A split on these variables therefore separated patients with and without a recorded measurement. Because calcitonin was recorded in 75% of patients with a synchronous DTC and in 31% of those without, and BMI and family history were also recorded more often in patients with the outcome within centres, with a similar but less precise pattern for the antibodies (Table S6), these variables acted as indicators of data completeness rather than as measurements. This interpretation is consistent with the missing-indicator analysis, which reproduced an AUROC of 0.75 (Table S8), and with the absence of discrimination within centres, across centres and over time in the revised analysis. In addition, the elastic-net AUROC of 0.74 reported in the submission had been computed on the complete-case training data, and the Youden threshold of 0.062 had been selected on the same out-of-bag predictions used to report sensitivity and specificity.

Table S1. Performance of the pre-operative risk-prediction models (estimates with 95% CI).

Measure	Random forest, nested CV	Elastic net (1-SE λ), nested CV	Elastic net (minimum λ), nested CV	Random forest, leave-one-centre-out	Random forest, temporal
AUROC	0.56 (0.47 to 0.65)	0.58 (0.48 to 0.67)	0.59 (0.50 to 0.67)	0.31 (0.24 to 0.38)	0.39 (0.28 to 0.51)
Average precision	0.087 (0.045 to 0.162)	0.077 (0.043 to 0.137)	0.071 (0.044 to 0.114)	0.035 (0.026 to 0.046)	0.063 (0.031 to 0.124)
Brier score	0.049 (0.038 to 0.060)	0.247 (0.245 to 0.250)	0.240 (0.235 to 0.244)	0.052 (0.041 to 0.063)	0.060 (0.040 to 0.080)
Scaled Brier score	0.003 (-0.223 to 0.228)	-4.067 (-4.111 to -4.022)	-3.912 (-4.005 to -3.819)	-0.063 (-0.296 to 0.170)	-0.042 (-0.386 to 0.302)
Calibration-in-the-large	-0.03 (-0.29 to 0.23)	-2.90 (-3.16 to -2.65)	-2.88 (-3.13 to -2.62)	-0.33 (-0.60 to -0.06)	0.31 (-0.11 to 0.72)
Calibration slope	0.45 (-0.21 to 1.11)	5.86 (-4.30 to 16.03)	1.67 (-0.47 to 3.82)	-1.48 (-1.99 to -0.97)	-0.37 (-0.82 to 0.09)
Expected/observed ratio	1.03 (0.80 to 1.33)	9.66 (7.50 to 12.45)	9.47 (7.35 to 12.21)	1.35 (1.04 to 1.76)	0.76 (0.50 to 1.14)
Threshold (median)	0.060	0.482	0.487	0.059	0.033
True positives	24	47	40	29	11
False positives	380	790	599	529	254
False negatives	37	14	21	32	18
True negatives	744	334	525	595	187
Sensitivity	0.40 (0.21 to 0.62)	0.76 (n.e.)	0.66 (0.40 to 0.85)	0.48 (0.36 to 0.60)	0.38 (0.22 to 0.57)
Specificity	0.67 (0.52 to 0.78)	0.27 (0.05 to 0.70)	0.46 (0.27 to 0.68)	0.53 (0.50 to 0.56)	0.42 (0.38 to 0.47)
Positive predictive value	0.060 (0.037 to 0.095)	0.056 (0.041 to 0.076)	0.063 (0.044 to 0.088)	0.052 (0.036 to 0.075)	0.042 (0.024 to 0.072)

Measure	Random forest, nested CV	Elastic net (1-SE λ), nested CV	Elastic net (minimum λ), nested CV	Random forest, leave-one-centre-out	Random forest, temporal
Negative predictive value	0.953 (0.934 to 0.967)	0.962 (n.e.)	0.962 (0.937 to 0.977)	0.949 (0.930 to 0.963)	0.912 (0.865 to 0.944)
Positive likelihood ratio	1.17 (0.77 to 1.79)	1.09 (0.88 to 1.36)	1.23 (0.95 to 1.60)	1.01 (0.77 to 1.32)	0.66 (0.41 to 1.07)
Negative likelihood ratio	0.90 (0.69 to 1.18)	0.73 (n.e.)	0.73 (0.46 to 1.18)	0.99 (0.78 to 1.27)	1.46 (1.07 to 1.99)
Balanced accuracy	0.53 (0.45 to 0.61)	0.53 (0.46 to 0.60)	0.56 (0.48 to 0.63)	0.50 (0.44 to 0.57)	0.40 (0.31 to 0.50)
Net benefit, threshold 5%	0.0053 (-0.0055 to 0.0160)	0.0016 (-0.0116 to 0.0147)	0.0016 (-0.0116 to 0.0147)	-0.0118 (-0.0215 to -0.0021)	-0.0029 (-0.0136 to 0.0078)
Net benefit, threshold 10%	0.0002 (-0.0050 to 0.0053)	-0.0539 (-0.0678 to -0.0400)	-0.0539 (-0.0678 to -0.0400)	-0.0092 (-0.0134 to -0.0049)	-0.0021 (-0.0097 to 0.0054)
Net benefit, threshold 15%	0.0002 (-0.0025 to 0.0029)	-0.1159 (-0.1306 to -0.1012)	-0.1159 (-0.1306 to -0.1012)	-0.0042 (-0.0056 to -0.0027)	-0.0009 (-0.0055 to 0.0038)
Net benefit, threshold 20%	0.0005 (-0.0014 to 0.0024)	-0.1857 (-0.2013 to -0.1700)	-0.1856 (-0.2013 to -0.1700)	-0.0008 (-0.0017 to 0.0000)	-0.0021 (-0.0042 to -0.0001)

Nested CV: 20-times repeated five-fold cross-validation (1185 patients, 61 events), estimates pooled with Rubin's rules; counts are means across repeats. Leave-one-centre-out: 22 held-out centres. Temporal: development 2017–2020, validation 2021–2023 (470 patients, 29 events). Prevalence 0.051 (temporal validation set 0.062). n.e., not estimable (sensitivity equal to 1 in some repeats; untransformed mean across repeats shown). Scaled Brier score derived from the pooled Brier score; negative values indicate predictions less accurate than the overall prevalence. The elastic net, fitted with balanced weights, assigns predicted probabilities close to 0.5 to all patients, which explains its calibration and net-benefit values.

Table S2. Contribution of individual predictors to the weighted random forest.

Predictor	Held-out permutation: decrease in AUROC (2.5th–97.5th percentile)	Ranked in top 3 (permutation)	LOCO: decrease in AUROC (95% CI)	randomForestSRC importance ×10³ (2.5th–97.5th percentile)	Ranked in top 3 (randomForestSRC)
Ab-TPO positivity	0.021 (0.002 to 0.036)	65%	0.019 (-0.007 to 0.045)	1.40 (0.30 to 4.52)	38%
Pre-operative clinical diagnosis	0.013 (-0.035 to 0.045)	45%	-0.000 (-0.029 to 0.029)	2.53 (0.17 to 6.23)	82%
Age	0.011 (-0.030 to 0.037)	40%	0.004 (-0.044 to 0.051)	0.05 (-1.76 to 1.55)	6%
Body-mass index	0.010 (-0.021 to 0.032)	35%	0.011 (-0.037 to 0.060)	0.45 (-0.63 to 3.04)	7%
Family history of thyroid disease	0.006 (-0.008 to 0.020)	20%	0.001 (-0.019 to 0.022)	0.16 (-0.54 to 0.82)	1%
EU-TIRADS category	0.004 (-0.021 to 0.031)	20%	-0.003 (-0.042 to 0.036)	1.87 (0.03 to 5.57)	67%
Nodule diameter on ultrasound	0.002 (-0.037 to 0.041)	40%	0.009 (-0.032 to 0.049)	0.37 (-0.52 to 2.84)	1%
Ab-Tg positivity	0.002 (-0.015 to 0.012)	20%	0.007 (-0.013 to 0.027)	1.77 (0.01 to 5.07)	32%
Cytology category (TIR)	0.001 (-0.022 to 0.021)	10%	-0.004 (-0.038 to 0.030)	2.11 (0.05 to 5.85)	64%
Irregular margins	-0.001 (-0.014 to 0.014)	5%	0.002 (-0.014 to 0.018)	0.35 (-0.17 to 1.71)	0%
Central vascularisation	-0.002 (-0.017 to 0.013)	0%	0.006 (-0.018 to 0.029)	0.01 (-0.60 to 1.08)	1%
Hypoechogenicity	-0.002 (-0.019 to 0.014)	0%	0.001 (-0.017 to 0.019)	0.31 (-0.19 to 1.88)	1%
Sex	-0.002 (-0.017 to 0.011)	0%	0.000 (-0.029 to 0.029)	0.08 (-0.48 to 1.59)	0%

Held-out permutation importance across 20 repeats; leave-one-covariate-out (LOCO) importance across 5 repeats; randomForestSRC Breiman–Cutler permutation importance (Brier score) across 100 training forests. Predictors ordered by held-out permutation importance.

Table S3. Hyperparameter configuration and tuning of the weighted random forest.

Variables per split (mtry)	Minimum terminal-node size	Times selected (of 100 training partitions)
2	10	18
2	3	41
2	30	20
3	10	3
3	3	9
3	30	8
5	30	1

Fixed settings: AUC-based splitting rule, 10 random split points, 1000 trees per imputed dataset (500 for tuning and for LOCO), class weights inversely proportional to the outcome frequencies in the training partition. Tuning criterion: out-of-bag AUROC within the training partition. Elastic net: $\alpha = 0.5$, λ by ten-fold cross-validation with the one-standard-error rule; median number of non-zero coefficients 3; no predictor retained in 16% of the training partitions.

Table S4. Centre-level recruitment, outcome prevalence and data completeness.

Centre	Patients	Synchronous DTC	Prevalence, % (95% CI)	Years	Total thyroid-ectomy, %	Report naming NIFTP, %	NIFTP-only with incompatible features	BRAF V600E (all patients)	Strict-histology subset, patients (DTC)	BMI missing, %	Cytology missing, %	Ab-TPO missing, %
C01	289	0	0.0 (0.0–1.3)	2017–2022	65	100	0	0	289 (0)	1	20	80
C02	261	0	0.0 (0.0–1.5)	2017–2022	71	20	48	0	52 (0)	7	0	91
C03	150	8	5.3 (2.7–10.2)	2019–2023	83	27	42	0	40 (8)	73	25	87
C04	91	3	3.3 (1.1–9.2)	2017–2023	86	21	50	31	19 (2)	84	4	92
C05	88	1	1.1 (0.2–6.2)	2019–2022	93	11	23	1	9 (0)	100	39	0
C06	70	9	12.9 (6.9–22.7)	2017–2022	91	91	6	1	59 (18)	1	7	1
C07	65	11	16.9 (9.7–27.8)	2017–2022	35	100	0	0	65 (11)	6	22	100
C08	36	8	22.2 (11.7–38.1)	2018–2022	81	97	1	0	34 (8)	14	0	11
C09	22	2	9.1 (2.5–27.8)	2017–2023	64	73	6	0	13 (2)	0	0	0
C10	19	6	31.6 (15.4–54.0)	2020–2022	74	89	0	0	17 (5)	0	21	5
C11	14	4	28.6 (11.7–54.6)	2019–2023	57	100	0	1	13 (3)	0	36	71
C12	14	0	0.0 (0.0–21.5)	2018–2023	43	100	0	0	14 (0)	100	0	100
C13	12	2	16.7 (4.7–44.8)	2017–2022	83	92	0	1	10 (1)	8	0	8
C14	11	3	27.3 (9.7–56.6)	2019–2022	91	100	0	0	11 (4)	100	18	100
C15	11	1	9.1 (1.6–37.7)	2019–2022	55	100	2	0	9 (1)	18	27	82
C16	9	0	0.0 (0.0–29.9)	2018–2022	67	67	1	0	6 (0)	100	0	100
C17	5	0	0.0 (0.0–43.4)	2023–2023	40	100	0	0	5 (0)	0	0	0
C18	7	0	0.0 (0.0–35.4)	2021–2023	57	71	0	0	5 (0)	0	0	100
C19	5	0	0.0 (0.0–43.4)	2019–2022	60	100	0	0	5 (0)	100	0	0
C20	2	1	50.0 (9.5–90.5)	2018–2023	100	100	0	0	2 (1)	0	0	0
C21	3	2	66.7 (20.8–93.9)	2022–2022	100	67	0	0	2 (2)	0	0	0
C22	1	0	0.0 (0.0–79.3)	2023–2023	0	100	0	0	1 (0)	0	0	0

Centres are coded C01–C22 in decreasing order of the number of registry records. Incompatible features: capsular invasion, angio-invasion, lymphatic invasion, tumour necrosis or extra-thyroidal extension coded in a NIFTP-only specimen. BRAF V600E: pre-operative cytology, patients with and without a synchronous DTC. Strict-histology subset (Section S1.10): report explicitly naming NIFTP, no feature incompatible with NIFTP for the index lesion, no BRAF V600E on cytology; synchronous DTCs after review of the reports in parentheses.

Table S5. Predictor dictionary.

Predictor	Case-report-form values	Coding in the models (reference)	Treated as missing	Notes
Sex	1 female, 2 male	Binary (female)	Blank	—
Age	Years at surgery	Continuous	Blank	Eligibility ≥18 years
Body-mass index	kg/m^2	Continuous	‘nd’; values <13 or >70	Pre-operative
Family history of thyroid disease	0 none, 1 benign thyroid disease, 2 thyroid malignancy, 3 risk factors	Nominal, 3 levels (none)	‘nd’	Code 3 not used
Pre-operative clinical diagnosis	1 goitre/multinodular goitre, 2 suspicious for malignancy, 3 indeterminate nodule, 4 benign nodule	Nominal, 4 levels (indeterminate)	Blank	Indication for surgery recorded by the treating team; code 2 labelled “malignant” in the form
EU-TIRADS category	1–5; 4A/4B used by one centre	Ordinal 1–5 (4A/4B → 4)	‘nd’, ‘x’	Dominant nodule
Cytology category	1 TIR 2, 2 TIR 3a, 3 TIR 3b, 4 TIR 4, 5 TIR 5	Ordinal 1–5	Blank	Bethesda II, III, IV, V, VI; dominant nodule
Echogenicity	1 hypoechoic, 2 hyperechoic, 3 anechoic, 4 isoechoic, 5 not determined	Binary: hypoechoic vs other	Code 5	Analysed as a 1–5 score in the submission
Vascularisation	1 peripheral, 2 central and peripheral, 3 not determined, 4 avascular	Binary: central and peripheral vs other	Code 3	Analysed as a 1–4 score in the submission
Sonographic margins	1 regular, 2 irregular, 3 not determined	Binary: irregular vs regular	Code 3	Analysed as a 1–3 score in the submission
Nodule diameter on ultrasound	mm, largest diameter of the dominant nodule	Continuous	‘nd’	First value when two were given
Calcitonin	0 normal, 1 elevated (local reference range); isolated numeric entries	Binary; excluded from models (4 elevated)	‘nd’	Reported as pg/mL in the submission
Ab-Tg	0 negative, 1 positive (local reference range)	Binary (negative)	‘nd’; one numeric entry (10)	‘<1’ and ‘<15’ coded negative
Ab-TPO	0 negative, 1 positive (local reference range)	Binary (negative)	‘nd’; numeric entries (20)	Reported as IU/mL in the submission

All variables refer to the pre-operative work-up preceding the index operation; dates of the individual examinations were not recorded. Harmonisation consisted of mapping the entries of each centre to the case-report-form codes listed; no interobserver standardisation of ultrasonography across centres was available. Pre-operative molecular testing (BRAF V600E, RAS) was not used as a predictor.

Table S6. Missing data by predictor, outcome and centre.

Predictor	Missing, n (%)	Missing in NIFTP only, %	Missing in NIFTP + DTC, %	p-value	Range across centres, %	Centres with all values missing	Variation explained by centre (McFadden R²)	OR of missingness for DTC, adjusted for centre (95% CI)
Sex	2 (0.2)	0.2	0.0	1	0–1	0	—	—
Age	3 (0.3)	0.3	0.0	1	0–1	0	—	—
Body-mass index	348 (29.4)	30.2	14.8	0.0091	0–100	5	0.64	0.19 (0.06–0.59)
Family history of thyroid disease	622 (52.5)	52.8	47.5	0.43	0–100	4	0.71	0.41 (0.20–0.86)
Pre-operative clinical diagnosis	1 (0.1)	0.1	0.0	1	0–8	0	—	—
EU-TIRADS category	137 (11.6)	11.8	6.6	0.3	0–100	1	0.24	0.72 (0.23–2.26)
Cytology category (TIR)	167 (14.1)	14.4	8.2	0.25	0–39	0	0.20	0.42 (0.16–1.16)
Hypoechogenicity	347 (29.3)	29.3	29.5	1	0–100	1	0.25	0.65 (0.32–1.32)
Central vascularisation	476 (40.2)	40.5	34.4	0.42	0–100	1	0.30	0.68 (0.34–1.36)
Irregular margins	464 (39.2)	39.8	27.9	0.079	0–100	2	0.33	0.61 (0.29–1.26)
Nodule diameter on ultrasound	16 (1.4)	1.4	0.0	1	0–18	0	—	—
Calcitonin elevated	790 (66.7)	69.0	24.6	5.8e-12	0–100	5	0.63	0.07 (0.02–0.27)
Ab-Tg positivity	790 (66.7)	68.1	39.3	9.1e-06	0–100	4	0.48	0.37 (0.14–1.02)
Ab-TPO positivity	816 (68.9)	70.3	42.6	1.4e-05	0–100	5	0.53	0.38 (0.13–1.13)

p-value: Fisher's exact test of missingness by outcome. R^2 and odds ratios from logistic models of the missingness indicator (shown for predictors with at least 20 missing values); an odds ratio below 1 indicates that the value was more often recorded in patients with a synchronous DTC.

Table S7. Case-level reconciliation of the synchronous DTCs: (A) the 61 coded by the centres; (B) 13 carcinomas described in the stored reports of patients coded as NIFTP-only.

#	Centre	Total thyroid-ectomy	Histotype	Recorded diameter	Diameter used, mm	Recorded stage	pT (AJCC 8th)	Multi-focal	Microscopic features (attributed to the carcinoma)	Nodes examined	pN	Potential management relevance	Source note
**A. Synchronous DTCs coded by the centres (primary analysis)**													
1	C03	yes	PTC, subtype not specified	3	15	pT2	pT1b	no	—	0	pNX	yes	text: PTC 15 mm left lobe + NIFTP 3 mm right lobe (diameters inverted in coded fields)
2	C03	yes	PTC, subtype not specified	5	5	pT1(a)	pT1a	no	—	0	pNX	no	

#	Centre	Total thyroid-ectomy	Histotype	Recorded diameter	Diameter used, mm	Recorded stage	pT (AJCC 8th)	Multi-focal	Microscopic features (attributed to the carcinoma)	Nodes examined	pN	Potential management relevance	Source note
3	C03	yes	PTC, subtype not specified	9	9	pT1(a)	pT1a	no	–	0	pNX	no	
4	C11	yes	PTC, subtype not specified	5	5	pT1b	pT1a	yes	–	0	pNX	no	
5	C15	yes	PTC, subtype not specified	6	6	pT1(a)	pT1a	no	–	1	pN0	no	
6	C14	yes	PTC, classic subtype	9	35	pT1(a)	pT2	yes	minimal ETE	0	pNX	yes	text: multifocal classic PTC, largest focus 35 mm, minimal ETE into perithyroidal soft tissue
7	C14	yes	PTC, classic subtype	9	18	pT1b	pT1b	yes	minimal ETE	1	pN0	yes	text: multifocal bilateral classic PTC, largest focus 18 mm, minimal ETE; median node negative
8	C14	yes	PTC, tall-cell subtype	9	9	pT1(a)	pT1a	no	–	1	pN0	yes	text: tall-cell PTC 9 mm, intrathyroidal; Delphian node negative
9	C08	yes	PTC, subtype not specified	6	6	pT1(a)	pT1a	no	–	5	pN0	no	
10	C08	no	PTC, subtype not specified	5	5	pT1(a)	pT1a	no	–	0	pNX	no	
11	C08	no	PTC, subtype not specified	7	7	pT1(a)	pT1a	no	–	1	pN0	no	
12	C07	no	PTC, subtype not specified	3	3	pT1(a)	pT1a	no	–	0	pNX	no	
13	C07	yes	PTC, subtype not specified	4	4	pT1(a)	pT1a	no	–	1	pN0	no	
14	C07	no	PTC, subtype not specified	6	6	pT1(a)	pT1a	no	–	0	pNX	no	
15	C07	yes	PTC, subtype not specified	5	5	pT1(a)	pT1a	no	–	0	pNX	no	
16	C07	no	PTC, subtype not specified	4	4	pT1(a)	pT1a	no	capsular invasion	0	pNX	no	
17	C07	yes	PTC, subtype not specified	7	7	pT1(a)	pT1a	no	minimal ETE	0	pNX	yes	
18	C07	no	PTC, subtype not specified	3	3	pT1(a)	pT1a	no	–	1	pN0	no	
19	C07	yes	PTC, subtype not specified	4	4	pT1(a)	pT1a	no	minimal ETE	4	pN0	yes	
20	C07	yes	PTC, subtype not specified	9	9	pT1(a)	pT1a	no	–	0	pNX	no	
21	C07	no	PTC, subtype not specified	8	8	pT1(a)	pT1a	no	minimal ETE	0	pNX	yes	
22	C07	yes	PTC, subtype not specified	6	6	pT1(a)	pT1a	no	minimal ETE	0	pNX	yes	
23	C04	yes	PTC, classic subtype	4	4	pT1(a)	pT1a	yes	–	0	pNX	no	
24	C04	yes	PTC, classic subtype	4	4	pT1(a)	pT1a	yes	angio-invasion, necrosis	1	pN0	yes	
25	C11	yes	Follicular carcinoma	9	9	pT1b	pT1a	yes	–	5	pN0	no	

#	Centre	Total thyroid-ectomy	Histotype	Recorded diameter	Diameter used, mm	Recorded stage	pT (AJCC 8th)	Multi-focal	Microscopic features (attributed to the carcinoma)	Nodes examined	pN	Potential management relevance	Source note
26	C09	yes	PTC, subtype not specified	3	3	pT1(a)	pT1a	no	—	1	pN0	no	
27	C11	no	PTC, subtype not specified	3	3	pT1(a)	pT1a	no	—	0	pNX	no	
28	C06	yes	PTC, classic subtype	3	3	pT1(a)	pT1a	no	—	8	pN0	no	text: classic papillary microcarcinoma 3 mm; perithyroidal nodes 0/8
29	C13	yes	PTC, subtype not specified	3	3	pT1(a)	pT1a	no	—	0	pNX	no	
30	C13	yes	PTC, subtype not specified	4	4	pT1(a)	pT1a	no	—	0	pNX	no	
31	C08	yes	PTC, subtype not specified	2	2	pT1(a)	pT1a	yes	—	4	pN0	no	
32	C20	yes	PTC, subtype not specified	6	6	pT1(a)	pT1a	no	—	0	pNX	no	
33	C10	yes	PTC, subtype not specified	4	4	pT1(a)	pT1a	no	—	4	pN0	no	
34	C10	yes	PTC, subtype not specified	4	4	pT1(a)	pT1a	no	—	0	pNX	no	
35	C21	yes	PTC, subtype not specified	6	6	pT1(a)	pT1a	no	—	2	pN0	no	
36	C21	yes	PTC, subtype not specified	4	4	pT1(a)	pT1a	no	—	2	pN0	no	
37	C03	yes	Follicular carcinoma	9	9	pT1(a)	pT1a	no	—	0	pNX	no	
38	C06	yes	Well-differentiated follicular tumour (microfollicular)	8	8	pT1(a)	pT1a	no	capsular invasion	1	pN0	no	report describes a single well-differentiated follicular microtumour (nuclear score 2) without separate mention of NIFTP; coded as carcinoma by the centre
39	C06	yes	PTC, classic subtype	1	1	pT1(a)	pT1a	no	—	0	pNX	no	text: classic papillary microcarcinoma 1 mm
40	C10	yes	Oncocytic carcinoma	3	3	pT1(a)	pT1a	no	—	0	pNX	no	
41	C04	yes	PTC, subtype not specified	9	9	pT3	pT1a	no	capsular invasion	7	pN0	no	report: carcinoma infiltrating the capsule; recorded pT3
42	C03	yes	PTC, subtype not specified	6	6	pT1(a)	pT1a	no	—	0	pNX	no	
43	C03	no	PTC, subtype not specified	3	3	pT3	pT1a	no	—	0	pNX	no	recorded pT3 without stated extra-thyroidal extension
44	C03	yes	PTC, subtype not specified	10	10	pT1(a)	pT1a	no	—	0	pNX	no	
45	C03	yes	PTC, subtype not specified	9	9	pT1(a)	pT1a	no	—	3	pN0	no	

#	Centre	Total thyroid-ectomy	Histotype	Recorded diameter	Diameter used, mm	Recorded stage	pT (AJCC 8th)	Multi-focal	Microscopic features (attributed to the carcinoma)	Nodes examined	pN	Potential management relevance	Source note
46	C09	yes	PTC, subtype not specified	4	4	pT1(a)	pT1a	no	—	1	pN0	no	
47	C11	yes	PTC, subtype not specified	6	6	pT1(a)	pT1a	no	—	0	pNX	no	
48	C06	yes	PTC, follicular subtype	1.5	1.5	pT1b	pT1a	yes	capsular invasion	4	pN0	no	text: two papillary microfoci, largest 1.5 mm (coded stage pT1b not supported)
49	C06	yes	PTC, follicular subtype	3	3	pT1b	pT1a	yes	—	8	pN0	no	text: multiple papillary microfoci, largest 3 mm (coded stage pT1b not supported)
50	C06	yes	PTC, subtype not specified	2	2	pT1(a)	pT1a	no	—	0	pNX	no	
51	C06	yes	PTC, subtype not specified	6	6	pT1(a)	pT1a	no	—	4	pN0	no	
52	C06	yes	PTC, subtype not specified	6	6	pT1(a)	pT1a	no	—	1	pN0	no	
53	C06	yes	PTC, subtype not specified	0.7	0.7	pT1(a)	pT1a	no	—	1	pN0	no	
54	C08	yes	PTC, subtype not specified	4	4	pT1(a)	pT1a	no	—	0	pNX	no	
55	C08	yes	PTC, subtype not specified	7	7	pT1(a)	pT1a	no	—	1	pN0	no	
56	C08	yes	PTC, subtype not specified	3	3	pT1b	pT1a	yes	—	2	pN0	no	
57	C08	no	PTC, subtype not specified	4	4	pT1(a)	pT1a	no	—	0	pNX	no	
58	C05	yes	PTC, follicular subtype	7	7	pT1(a)	pT1a	no	minimal ETE, capsular invasion	0	pNX	yes	report: breached capsule attributed to the lesion labelled NIFTP; excluded from the strict-histology subset
59	C10	yes	PTC, subtype not specified	10	10	pT1(a)	pT1a	no	—	0	pNX	no	
60	C10	yes	PTC, subtype not specified	6	6	pT1(a)	pT1a	no	—	0	pNX	no	
61	C10	no	PTC, subtype not specified	5	5	pT1(a)	pT1a	no	—	0	pNX	no	
**B. Carcinomas described in the stored report of patients coded as NIFTP-only (adjudicated outcome)**													
B1	C14	yes	Follicular carcinoma, encapsulated angio-invasive	—	47	pT3	pT3a	no	angio-invasion (<4 vessels)	0	pNX	yes	NIFTP in the contralateral lobe; meets criteria mandating a more extensive operation (total thyroidectomy performed)
B2	C06	yes	PTC, classic and follicular subtype	—	4	—	pT1a	yes	lymphatic invasion (coded)	4	pN0	no	two foci, 4 and 1 mm

#	Centre	Total thyroid-ectomy	Histotype	Recorded diameter	Diameter used, mm	Recorded stage	pT (AJCC 8th)	Multi-focal	Microscopic features (attributed to the carcinoma)	Nodes examined	pN	Potential management relevance	Source note
B3	C06	yes	PTC, classic	—	2	—	pT1a	no	—	5	pN0	no	just under 2 mm
B4	C06	yes	Papillary microtumour	—	0.5	—	pT1a	no	—	1	pN0	no	
B5	C06	yes	Papillary microtumour, infiltrative pattern	—	1	—	pT1a	no	—	3	pN0	no	
B6	C06	no	Papillary microtumour	—	—	—	pT1a	no	—	2	pN0	no	size not stated; a few neoplastic follicles
B7	C06	yes	Papillary neoplasm (microfocus)	—	0.4	—	pT1a	no	—	0	pNX	no	
B8	C06	yes	Papillary microtumour, follicular subtype, focally infiltrative	—	1	—	pT1a	no	—	2	pN0	no	
B9	C06	yes	PTC, follicular subtype	—	4	—	pT1a	yes	—	0	pNX	no	four further submillimetric foci
B10	C06	yes	PTC, follicular architecture	—	0.3	—	pT1a	yes	capsular invasion (coded)	4	pN0	no	second focus of a single follicle
B11	C06	yes	Papillary microtumour, classic	—	1	—	pT1a	yes	—	3	pN0	no	two foci of 1 mm
B12	C06	yes	Papillary microtumour	—	<1	—	pT1a	no	—	0	pNX	no	
B13	C06	yes	Papillary microtumour	—	<1	—	pT1a	no	—	0	pNX	no	

Part B: reports that explicitly named NIFTP and described a separate carcinoma, identified by review of the free text (Section S1.10); diameters and nodes as described in the report (—, not recorded). Part A: diameters in mm (two entries, 1.5 and 0.7, are reported as recorded). Where the stored report gave the size of the largest carcinoma focus, this value was used (source note). pT is size-derived according to the AJCC eighth edition (minimal extra-thyroidal extension not T-defining); pN0 when at least one node was examined and free of metastasis, otherwise pNX. Potential management relevance: diameter >1 cm, minimal extra-thyroidal extension, vascular invasion or tall-cell subtype.

Table S8. Sensitivity analyses.

Scenario	Model	Patients	Events	AUROC (95% CI)	Average precision	No-skill reference	Calibration slope (95% CI)	E/O	Sensitivity	Specificity
Primary analysis (20 repeats)	RF	1185	61	0.56 (0.47 to 0.65)	0.087	0.051	0.45 (−0.21 to 1.11)	1.03	0.40	0.67
Primary analysis (20 repeats)	EN	1185	61	0.58 (0.48 to 0.67)	0.077	0.051	5.86 (−4.30 to 16.03)	9.66	0.76	0.27
Native randomForestSRC imputation (nested)	RF	1185	61	0.57 (0.48 to 0.66)	0.115	0.051	0.39 (-0.10 to 0.87)	1.25	0.57	0.49
Outcome in training-data imputation	RF	1185	61	0.58 (0.48 to 0.66)	0.091	0.051	0.57 (-0.14 to 1.29)	1.03	0.43	0.7

Scenario	Model	Patients	Events	AUROC (95% CI)	Average precision	No-skill reference	Calibration slope (95% CI)	E/O	Sensitivity	Specificity
Outcome in training-data imputation	EN	1185	61	0.61 (0.52 to 0.69)	0.079	0.051	2.01 (-1.02 to 5.04)	9.46	0.63	0.53
Missing-indicator coding	RF	1185	61	0.75 (0.69 to 0.81)	0.154	0.051	1.21 (0.76 to 1.65)	1	0.75	0.65
Missing-indicator coding	EN	1185	61	0.58 (0.46 to 0.69)	0.084	0.051	1.33 (-2.01 to 4.66)	9.43	0.69	0.44
Reduced model (8 predictors <30% missing)	RF	1185	61	0.53 (0.45 to 0.61)	0.083	0.051	0.15 (-0.24 to 0.54)	1.06	0.26	0.75
Reduced model (8 predictors <30% missing)	EN	1185	61	0.50 (0.42 to 0.57)	0.096	0.051	n.e.	9.71	1	0
No class weighting	RF	1185	61	0.57 (0.49 to 0.65)	0.092	0.051	0.53 (-0.09 to 1.16)	1.03	0.41	0.67
No class weighting	EN	1185	61	0.49 (0.43 to 0.56) [a]	0.229	0.051	-3.88 (-37.00 to 29.24)	1	0.97	0.03
Total thyroidectomy only	RF	863	50	0.54 (0.42 to 0.65)	0.095	0.058	0.28 (-0.52 to 1.09)	1.04	0.42	0.61
Total thyroidectomy only	EN	863	50	0.51 (0.40 to 0.62)	0.069	0.058	2.10 (-21.93 to 26.13)	8.62	0.99	0.01
Centres with ≥1 recorded DTC	RF	594	61	0.53 (0.44 to 0.62)	0.144	0.103	0.22 (-0.55 to 0.99)	1.03	0.33	0.67
Centres with ≥1 recorded DTC	EN	594	61	0.50 (0.41 to 0.59)	0.143	0.103	n.e.	4.87	1	0
Adjudicated outcome (review of reports)	RF	1185	74	0.62 (0.54 to 0.69)	0.129	0.062	0.91 (0.00 to 1.81)	1.01	0.47	0.71
Adjudicated outcome (review of reports)	EN	1185	74	0.65 (0.57 to 0.72)	0.110	0.062	4.89 (0.14 to 9.65)	7.88	0.71	0.50
Strict-histology subset (adjudicated outcome)	RF	680	66	0.70 (0.62 to 0.78)	0.209	0.097	1.34 (0.59 to 2.09)	1.00	0.63	0.66
Strict-histology subset (adjudicated outcome)	EN	680	66	0.67 (0.57 to 0.75)	0.169	0.097	2.80 (0.16 to 5.44)	4.98	0.68	0.53

Ten repeats of five-fold cross-validation for each sensitivity analysis. EN, elastic-net logistic regression (balanced weights except in the unweighted scenario); RF, weighted random forest; E/O, expected-to-observed ratio; n.e., not estimable (constant or near-constant predictions). [a] No predictor retained in any training partition; discrimination and calibration estimates are not interpretable. Adjudicated outcome: carcinomas described in the stored reports but not coded by the centres added to the outcome (Section S1.10; Table S7, part B). Restricted outcome of potential management relevance (10 events), primary random-forest predictions: AUROC 0.55 (0.34 to 0.75). Within-centre concordance of the random-forest predictions (cluster-bootstrap 95% CI): primary 0.48 (0.37–0.54), missing-indicator 0.61 (0.34–0.84), total thyroidectomy 0.47 (0.31–0.56), centres with ≥1 DTC 0.49 (0.34–0.61), adjudicated outcome 0.48 (0.41–0.55), strict-histology subset 0.59 (0.52–0.75). Strict-histology subset, leave-one-centre-out validation: pooled AUROC 0.44 (0.37 to 0.51); random-effects summary of centre-specific AUROC 0.62 (0.52 to 0.71; 7 centres).

Table S9. Leave-one-centre-out validation of the weighted random forest.

Held-out centre	Patients	Observed events	Expected events	Observed/expected	AUROC (95% CI)
C01	289	0	20.1	0.00	—
C02	261	0	23.1	0.00	—
C03	150	8	11.5	0.70	0.39 (0.20–0.63)
C04	91	3	5.8	0.52	0.41 (0.14–0.75)
C05	88	1	6.0	0.17	—
C06	70	9	3.9	2.33	0.40 (0.22–0.61)
C07	65	11	3.2	3.44	0.50 (0.30–0.69)
C08	36	8	1.6	4.91	0.53 (0.28–0.76)
C09	22	2	1.4	1.42	—
C10	19	6	0.9	6.88	0.67 (0.33–0.89)
C11	14	4	0.8	4.83	0.50 (0.18–0.82)
C12	14	0	0.7	0.00	—
C13	12	2	0.5	3.79	—
C14	11	3	0.7	4.26	0.58 (0.20–0.89)
C15	11	1	0.6	1.61	—
C16	9	0	0.5	0.00	—
C17	5	0	0.3	0.00	—
C18	7	0	0.4	0.00	—
C19	5	0	0.3	0.00	—
C20	2	1	0.0	20.03	—
C21	3	2	0.2	12.78	—
C22	1	0	0.1	0.00	—

AUROC shown for centres with at least three patients with and three without the outcome. Random-effects summaries: AUROC 0.48 (95% CI 0.38–0.57; 8 centres); observed/expected ratio 1.37 (0.84–2.21), 95% prediction interval 0.24–7.84, I^2 70% (22 centres, 0.5 added to observed and expected events). Pooled leave-one-centre-out AUROC 0.31 (0.24–0.38).

Table S10. Reconciliation with the analysis of the original submission (1185 eligible patients).

Step	Predictor coding	Predictors	Imputation	Evaluation	AUROC
A. Submitted analysis, reproduced	Original	14	randomForestSRC, full data (outcome available)	Out-of-bag	0.77
B. A with ‘not determined’ codes set to missing	Original, ‘not determined’ missing	14	As A	Out-of-bag	0.76
C. A without calcitonin	Original	13	As A	Out-of-bag	0.72
D. Corrected coding, calcitonin retained	Corrected	14	As A	Out-of-bag	0.77
E. Corrected coding, calcitonin excluded	Corrected	13	As A	Out-of-bag	0.61
F. E with outcome-free imputation	Corrected	13	Chained equations, full data, outcome excluded	Out-of-bag	0.53
G. A nested in cross-validation	Original	14	randomForestSRC within training folds	5-fold CV, 3 repeats	0.71–0.73

Step	Predictor coding	Predictors	Imputation	Evaluation	AUROC
H. Revised primary analysis	Corrected	13	Chained equations within training folds, outcome excluded	Nested 5-fold CV, 20 repeats	0.56 (0.47–0.65)
I. H with missing-indicator coding	Corrected + missingness indicators	13	Median/mode within training folds	Nested 5-fold CV, 10 repeats	0.75 (0.69–0.81)

Steps A–F use the hyperparameters of the submission (3000 trees, mtry 3, node size 3, 10 split points, AUC splitting, inverse-frequency class weights, five imputation iterations). Calcitonin was observed as 0 in 99% of measured patients and imputed with non-zero values in all patients without a measurement, so that it identified the patients whose calcitonin had not been recorded (Section S3).

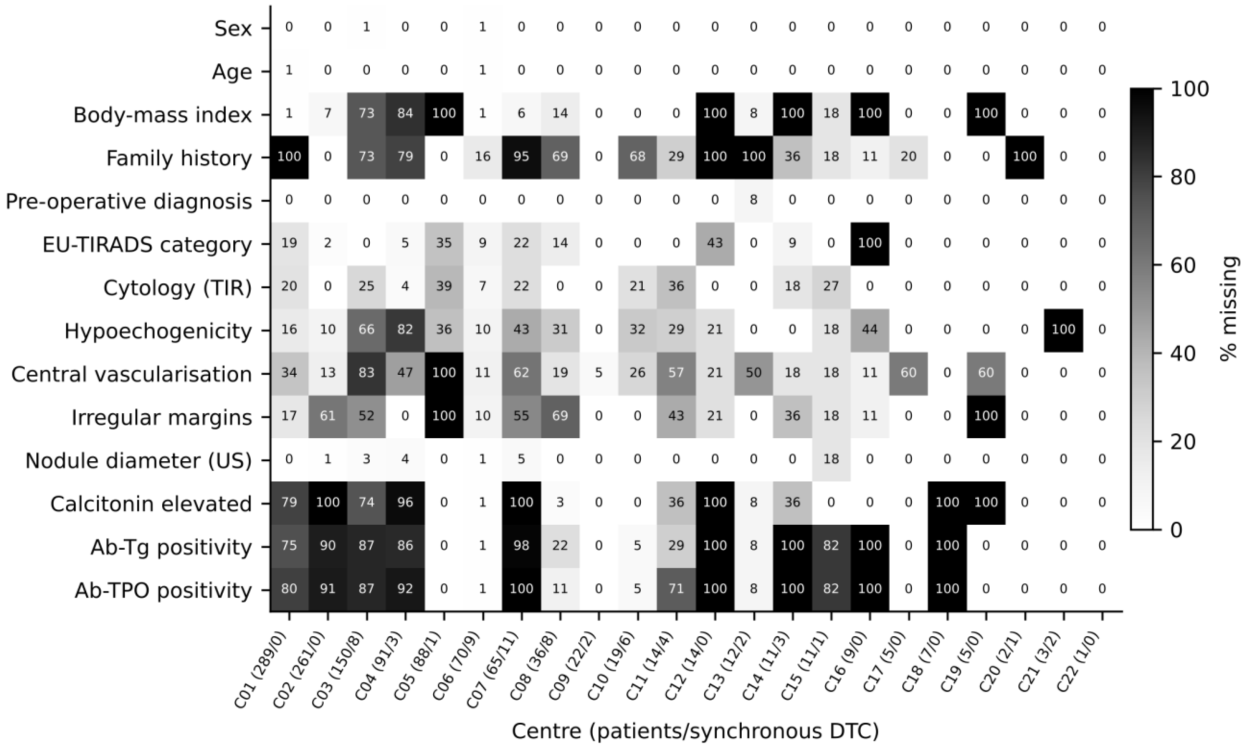

Figure S1. Percentage of missing values by predictor and centre. Centre labels give the number of patients and of synchronous DTCs.

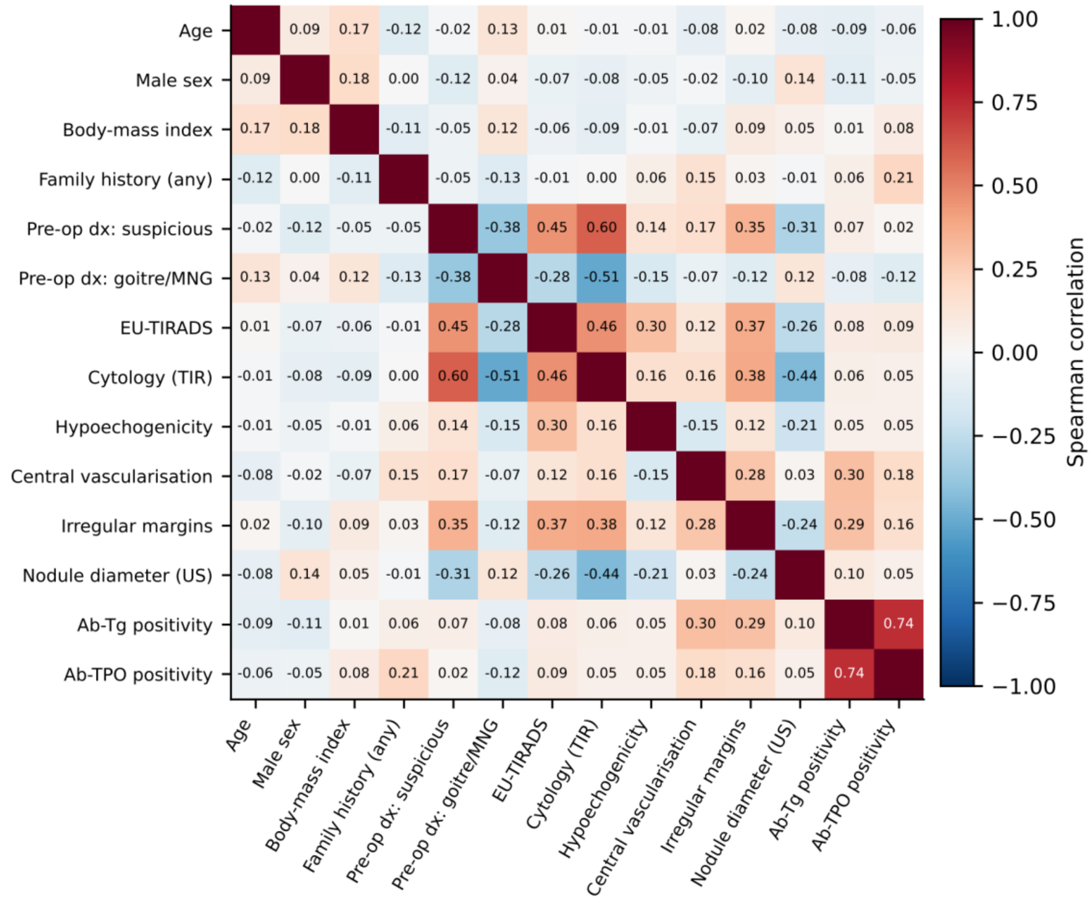

Figure S2. Spearman correlation matrix of the pre-operative predictors (pairwise complete observations; binary predictors coded 0/1; the pre-operative diagnosis represented by indicators of suspicious nodule and goitre).

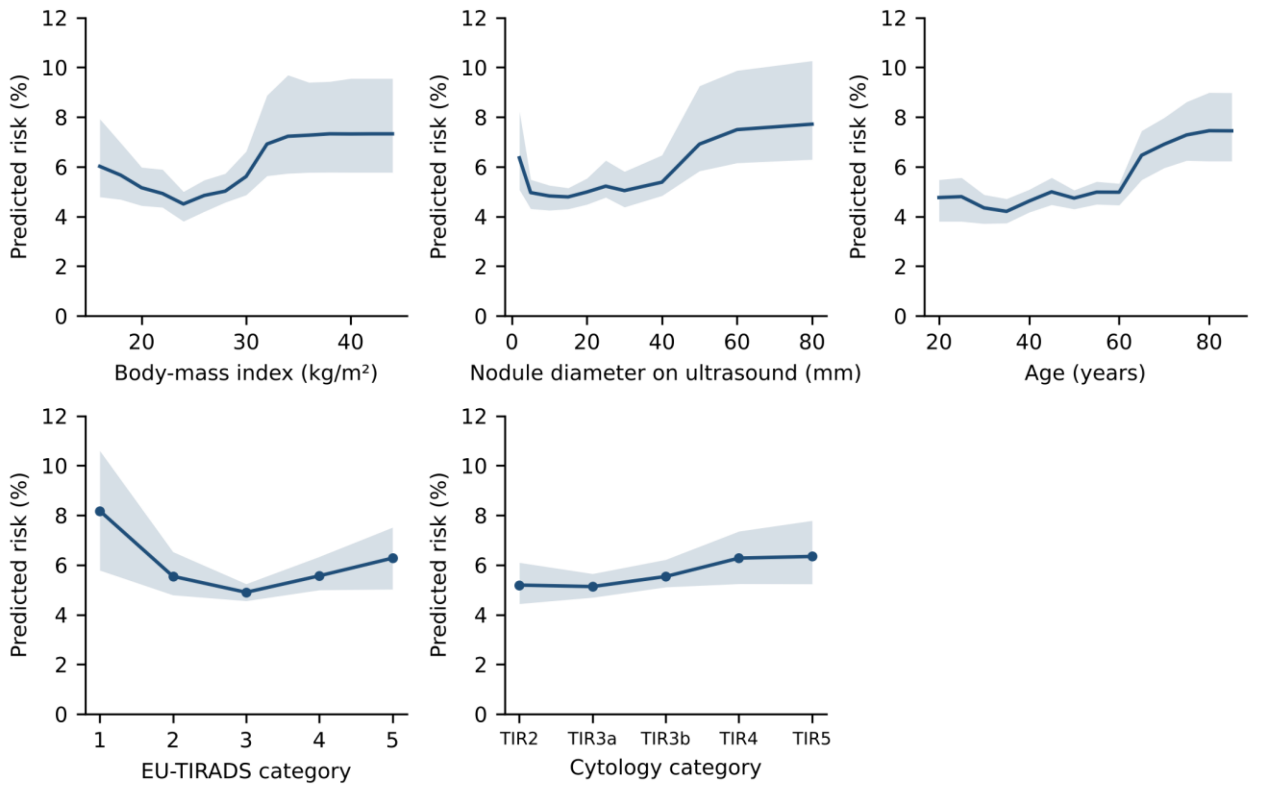

Figure S3. Partial-dependence functions of the weighted random forest for body-mass index, nodule diameter, age, EU-TIRADS category and cytology category: median predicted risk (line) and 2.5th–97.5th percentiles (band) across the 100 training partitions of the nested cross-validation.

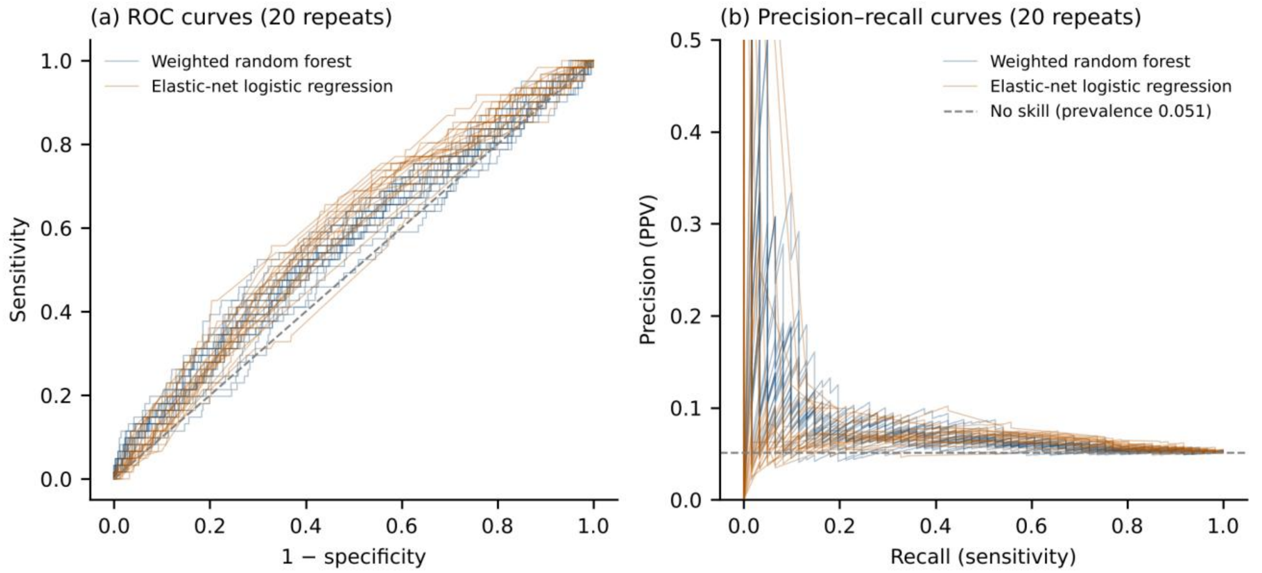

Figure S4. Receiver operating characteristic (a) and precision–recall (b) curves of the out-of-fold predictions of the weighted random forest and of the elastic-net logistic regression in each of the 20 repeats of nested cross-validation. The dashed line in (b) is the no-skill reference (outcome prevalence).

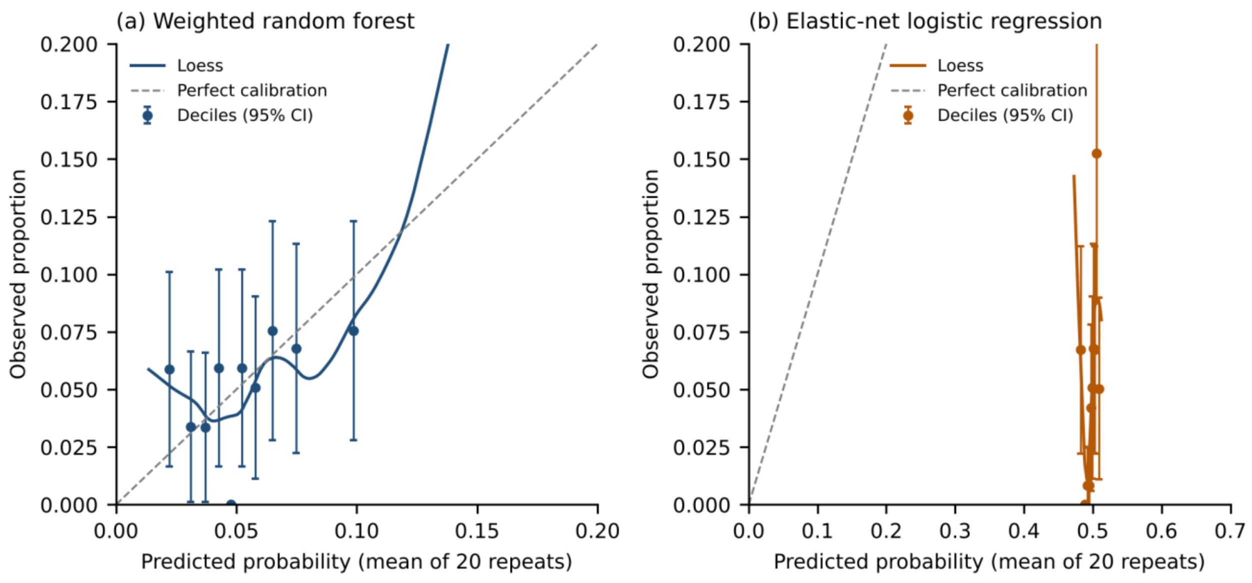

Figure S5. Calibration of the cross-validated predictions (mean of 20 repeats per patient): observed proportion by decile of predicted risk with 95% CI and loess smoother. Note the different horizontal scales: the elastic net, fitted with balanced weights, predicts risks close to 0.5 for all patients.

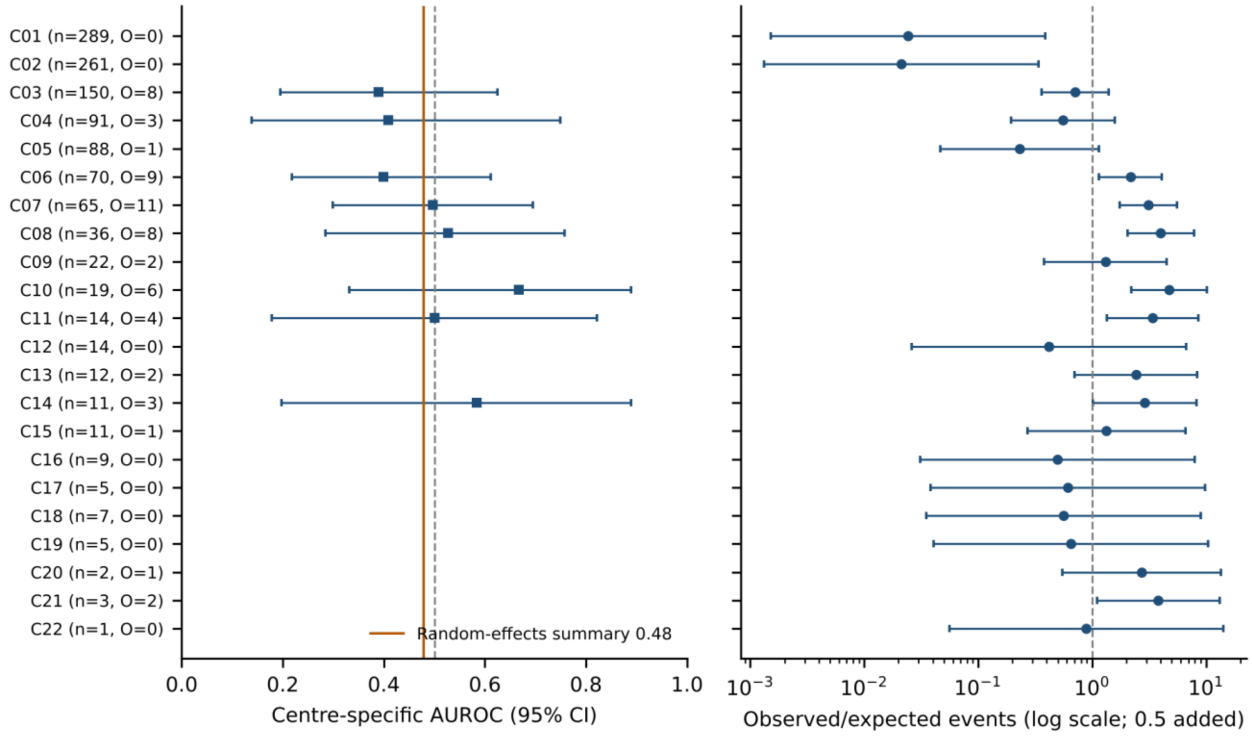

Figure S6. Leave-one-centre-out validation: centre-specific AUROC (left; centres with at least three patients with and without the outcome) and observed-to-expected ratio of synchronous DTCs (right; log scale, 0.5 added to observed and expected counts). The vertical line on the left marks the random-effects summary AUROC.
